# Five million years of Antarctic Circumpolar Current strength variability

**DOI:** 10.1038/s41586-024-07143-3

**Published:** 2024-03-27

**Authors:** Frank Lamy, Gisela Winckler, Helge W. Arz, Jesse R. Farmer, Julia Gottschalk, Lester Lembke-Jene, Jennifer L. Middleton, Michèlle van der Does, Ralf Tiedemann, Carlos Alvarez Zarikian, Chandranath Basak, Anieke Brombacher, Levin Dumm, Oliver M. Esper, Lisa C. Herbert, Shinya Iwasaki, Gaston Kreps, Vera J. Lawson, Li Lo, Elisa Malinverno, Alfredo Martinez-Garcia, Elisabeth Michel, Simone Moretti, Christopher M. Moy, Ana Christina Ravelo, Christina R. Riesselman, Mariem Saavedra-Pellitero, Henrik Sadatzki, Inah Seo, Raj K. Singh, Rebecca A. Smith, Alexandre L. Souza, Joseph S. Stoner, Maria Toyos, Igor M. Venancio P. de Oliveira, Sui Wan, Shuzhuang Wu, Xiangyu Zhao

**Affiliations:** 1 Alfred Wegener Institute (AWI) Helmholtz Centre for Polar and Marine Research, Bremerhaven, Germany; 2 MARUM – Center for Marine Environmental Sciences, University of Bremen, Bremen, Germany; 3 Lamont-Doherty Earth Observatory, Climate School, Columbia University, Palisades, NY USA; 4 Department of Earth and Environmental Sciences, Columbia University, New York, NY USA; 5 Leibniz Institute for Baltic Sea Research Warnemünde, Rostock, Germany; 6 School for the Environment, University of Massachusetts Boston, Boston, MA USA; 7 Institute of Geosciences, Kiel University, Kiel, Germany; 8 International Ocean Discovery Program, Texas A&M University, College Station, TX USA; 9 Department of Earth Sciences, University of Delaware, Newark, DE USA; 10 Department of Earth & Planetary Sciences, Yale University, New Haven, CT USA; 11 Berlin, Germany; 12 School of Marine and Atmospheric Sciences, Stony Brook University, Stony Brook, NY USA; 13 Research and Development Center for Global Change, Japan Agency for Marine-Earth Science and Technology (JAMSTEC), Yokosuka, Japan; 14 Department of Earth and Planetary Sciences, Rutgers, The State University of New Jersey, New Brunswick, NJ USA; 15 Department of Geosciences, National Taiwan University, Taipei, Taiwan; 16 Department of Earth and Environmental Sciences, University of Milano-Bicocca, Milan, Italy; 17 Climate Geochemistry Department, Max Planck Institute for Chemistry (MPIC), Mainz, Germany; 18 Laboratoire des Sciences du Climat et de l’Environnement (LSCE), Institut Pierre Simon Laplace (IPSL), CNRS-CEA-UVSQ, Gif-sur-Yvette, France; 19 Department of Geology, University of Otago, Dunedin, New Zealand; 20 Ocean Sciences Department, University of California, Santa Cruz, Santa Cruz, CA USA; 21 School of the Environment, Geography and Geosciences, University of Portsmouth, Portsmouth, UK; 22 Global Ocean Research Center, Korea Institute of Ocean Science and Technology (KIOST), Busan, Republic of Korea; 23 School of Earth, Ocean and Climate Sciences, Indian Institute of Technology Bhubaneswar, Bhubaneswar, India; 24 Department of Geosciences, University of Massachusetts Amherst, Amherst, MA USA; 25 Department of Geology, Federal University of Rio de Janeiro, Rio de Janeiro, Brazil; 26 College of Earth, Ocean, and Atmospheric Sciences, Oregon State University, Corvallis, OR USA; 27 Postgraduate Program in Geochemistry, Department of Geochemistry, Institute of Chemistry, Fluminense Federal University, Niterói, Brazil; 28 South China Sea Institute of Oceanology, Chinese Academy of Sciences, Guangzhou, China; 29 Institute of Earth Sciences, University of Lausanne, Lausanne, Switzerland; 30 Geoscience Group, National Institute of Polar Research, Tokyo, Japan

**Keywords:** Palaeoceanography, Palaeoclimate

## Abstract

The Antarctic Circumpolar Current (ACC) represents the world’s largest ocean-current system and affects global ocean circulation, climate and Antarctic ice-sheet stability^1–3^. Today, ACC dynamics are controlled by atmospheric forcing, oceanic density gradients and eddy activity^4^. Whereas palaeoceanographic reconstructions exhibit regional heterogeneity in ACC position and strength over Pleistocene glacial–interglacial cycles^5–8^, the long-term evolution of the ACC is poorly known. Here we document changes in ACC strength from sediment cores in the Pacific Southern Ocean. We find no linear long-term trend in ACC flow since 5.3 million years ago (Ma), in contrast to global cooling^9^ and increasing global ice volume^10^. Instead, we observe a reversal on a million-year timescale, from increasing ACC strength during Pliocene global cooling to a subsequent decrease with further Early Pleistocene cooling. This shift in the ACC regime coincided with a Southern Ocean reconfiguration that altered the sensitivity of the ACC to atmospheric and oceanic forcings^11–13^. We find ACC strength changes to be closely linked to 400,000-year eccentricity cycles, probably originating from modulation of precessional changes in the South Pacific jet stream linked to tropical Pacific temperature variability^14^. A persistent link between weaker ACC flow, equatorward-shifted opal deposition and reduced atmospheric CO_2_ during glacial periods first emerged during the Mid-Pleistocene Transition (MPT). The strongest ACC flow occurred during warmer-than-present intervals of the Plio-Pleistocene, providing evidence of potentially increasing ACC flow with future climate warming.

## Abstract (web summary)

The strength of the Antarctic Circumpolar Current, as traced in sediment cores from the Pacific Southern Ocean, shows no linear long-term trend over the past 5.3 Myr; instead, the strongest flow occurs consistently in warmer-than-present intervals.

## Main

The strong eastward flow of the ACC represents the world’s largest current system. It connects all three main basins of the global ocean and therefore integrates, and responds to, climate signals around the world^3^. The ACC reaches abyssal water depths and connects deep, intermediate and shallow ocean circulation^3^. The system of oceanic fronts across the ACC is associated with upward shoaling of density surfaces towards the south, upwelling of deep waters, the formation of intermediate water masses and steep upper-ocean gradients^15,16^. Through this linkage of the shallow and deep ocean, the ACC plays a critical role in the Southern Ocean carbon cycle and changes in atmospheric CO_2_ (ref. ^4^). The strength and position of the ACC and its associated oceanic fronts are controlled by wind stress, interaction of flow with the deep-ocean bathymetry and buoyancy forcing^4^. The Southern Westerly Winds (SWW), as the integrated wind stress across the entire circumpolar belt, drive northward transport of surface water in the Ekman layer, producing downwelling to the north and upwelling south of the wind belt. The SWW produce eastward geostrophic flow and form a vigorous eddy field interacting with rough bottom topography along the path of the ACC, thereby partly balancing the forcing at the sea surface^4^. Buoyancy forcing is controlled by heat and freshwater inputs that affect the density structure of the ACC and is thought to be equally important for ACC strength as the winds^4^.

During the past decades, warming around Antarctica (that is, south of the ACC) has been shown to be delayed compared with global atmospheric warming, yet a speed-up of the subantarctic ACC is observed in response to greenhouse-gas forcing^17^. This contributes to build-up of heat in the subtropics, north of the ACC, connected to poleward-shifting large-scale ocean gyres that are critical for anthropogenic heat uptake and transport^17,18^. Atmosphere–ocean interactions across the ACC also affect the extent and stability of the Antarctic cryosphere by altering the advection of comparably warm water masses, such as Circumpolar Deep Water, towards marine-based ice-sheet sections that are sensitive to subglacial melting^19^.

Sediment records of Pleistocene ACC strength in the Southeast Pacific sector of the Southern Ocean and the Drake Passage document a common pattern of reduced ACC flow during glacials^5,8^, including millennial-scale variations in phase with Antarctic palaeotemperature records^5,20^. On the other hand, small opposite variations in ACC strength are documented in sediment records across the southern ACC east of the Drake Passage in the Scotia Sea^7^, whereas stronger glacial ACC flow is reconstructed in the Indian Ocean sector^6^ and within the deep western boundary current east of New Zealand^21^. These observations highlight potential regional and meridional heterogeneity of ACC flow over Pleistocene glacial–interglacial cycles. Thus, an explicit north–south transect across the ACC zones in the pelagic Southern Ocean is important to assess overall ACC fluctuations.

Existing ACC strength records during the Pliocene are fragmentary^11^. Reconstructions of Southern Hemisphere meridional sea surface temperature (SST) gradients indicate an overall strengthening of the atmospheric circulation and plausibly imply an enhancement of the largely wind-driven ACC over the Pliocene and Early Pleistocene^9^. Moreover, Pliocene changes in tropical palaeoclimates (for example, the Asian monsoon^22^) and tropical Pacific zonal SST trends^23^ might affect Pliocene SWW intensity and thereby the atmospheric forcing of ACC strength. The Plio-Pleistocene evolution of these ACC drivers highlights the need for continuous ACC proxy records extending into the Pliocene to better understand the variability of ACC strength and associated ocean–atmosphere processes during warmer-than-present time periods.

**Fig. 1:**
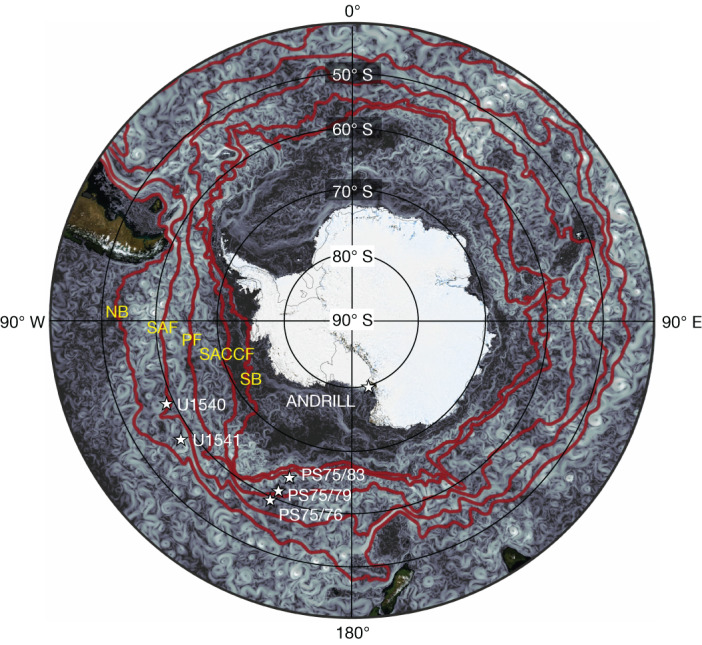
Visualization of the modern ACC. Shown is the simulated ocean velocity at 100-m water depth (blue = weak; white = strong). Model: FESOM2 (Finite-volumE Sea ice–Ocean Model, formulated on unstructured mesh; https://fesom.de/); setup: ROSSBY4.2; simulations: Dmitry Sein (AWI); visualization: Nikolay Koldunov (AWI). ACC fronts as derived from satellite altimetry^16^. From north to south: NB, north boundary; SAF, Subantarctic Front; PF, polar front; SACCF, southern ACC front; SB, southern boundary. Core and drilling locations are marked by white stars.

**Extended Data Fig. 1:**
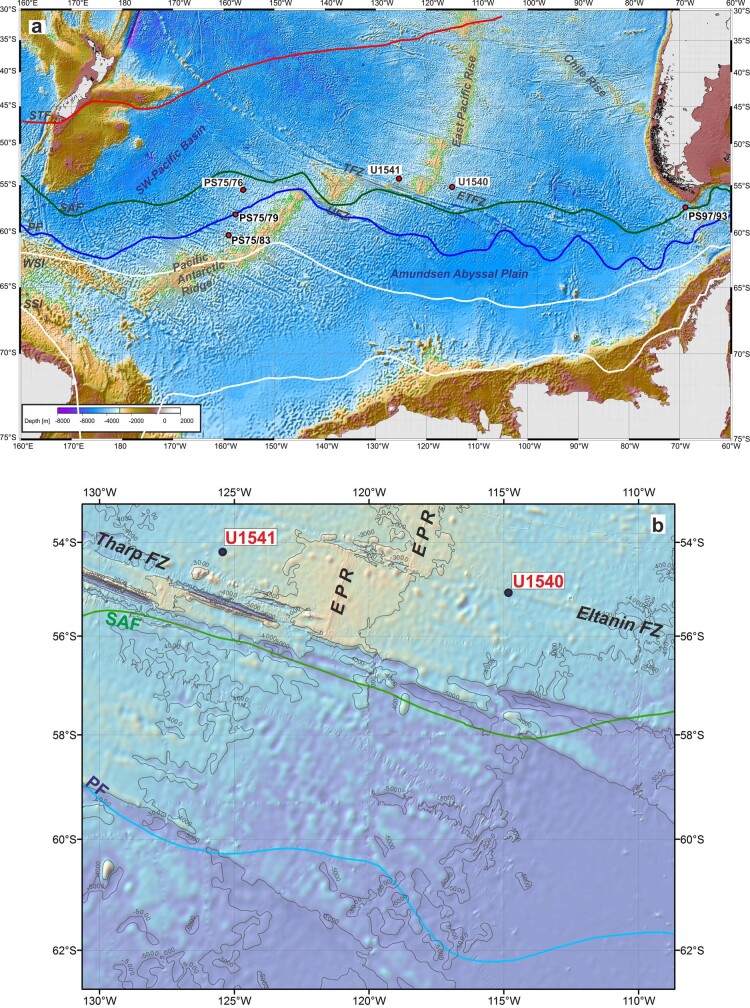
Bathymetric maps. **a**, South Pacific overview with location of all study sites, **b**, Detail of the central South Pacific with IODP sites. Besides regional topographic features, oceanic fronts after ref. ^55^ are also indicated. The yellow crossing lines are seismic surveys for IODP Expedition 383 (ref. ^54^). FZ, fracture zone.

**Extended Data Fig. 2:**
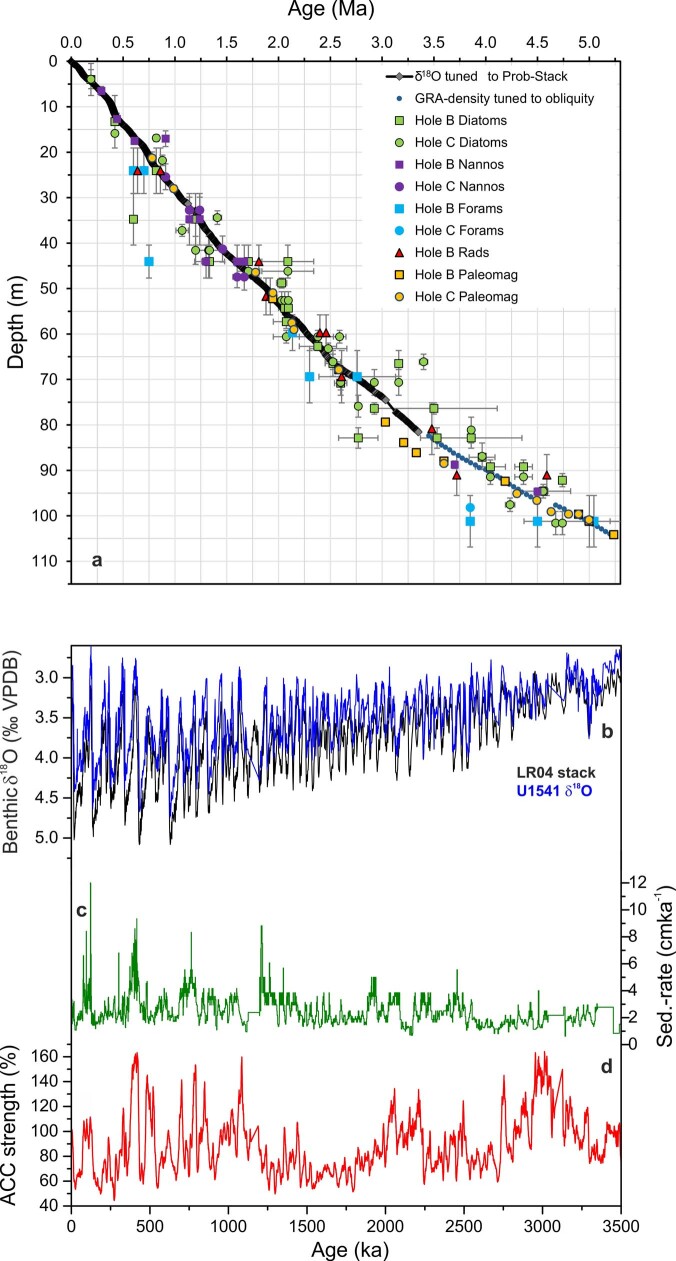
Stratigraphic background for IODP Site U1541. **a**, Age–depth plot for the Pliocene and Pleistocene sedimentary sequence at IODP Site U1541 compared with biostratigraphic and palaeomagnetic tie points^25^. Vertical error bars represent the depth range of each stratigraphic marker (for example, the biostratigraphic ages were determined on core catchers during the expedition). Horizontal error bars represent age uncertainties adopted from the best-fit-interval output of the constrained optimization analysis of Southern Hemisphere biostratigraphic datasets. Palaeomagnetic ages are based on the geomagnetic timescale GTS2012. Further details are discussed in the methods section of ref. ^54^. **b**, Benthic δ^18^O record from IODP Site U1541 tuned to the Prob-stack^26,56^, shown here in comparison with the LR04 stack^10^. **c**, Sedimentation-rate record at Site U1541. **d**, ACC strength record at IODP Site U1541.

**Extended Data Fig. 3:**
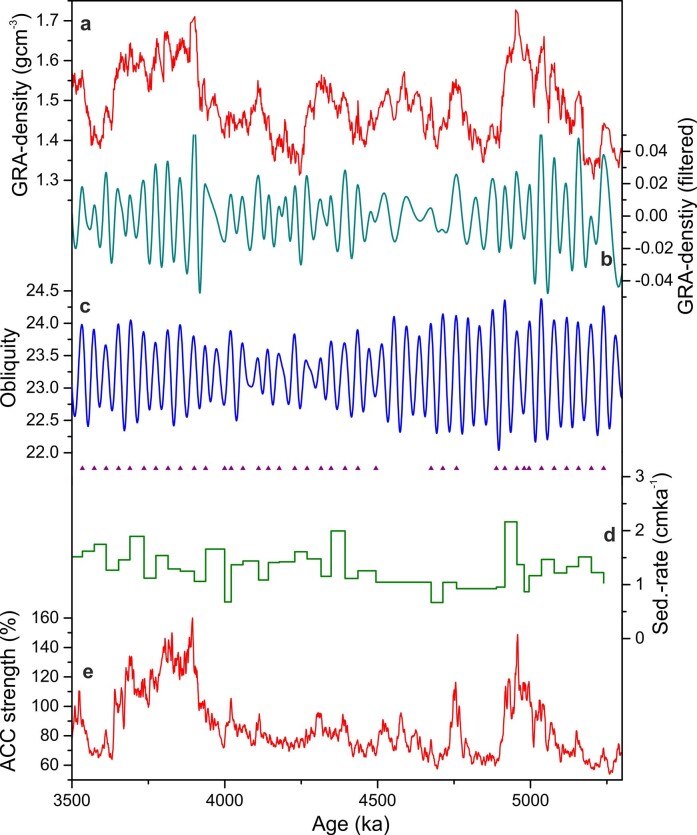
Pliocene stratigraphy for IODP Site U1541 based on orbital tuning. **a**, GRA density record. **b**, Obliquity (about 40 kyr) filtered GRA density record. **c**, Obliquity reference record with tuning points. **d**, Sedimentation-rate record. **e**, ACC strength record.

**Extended Data Fig. 4:**
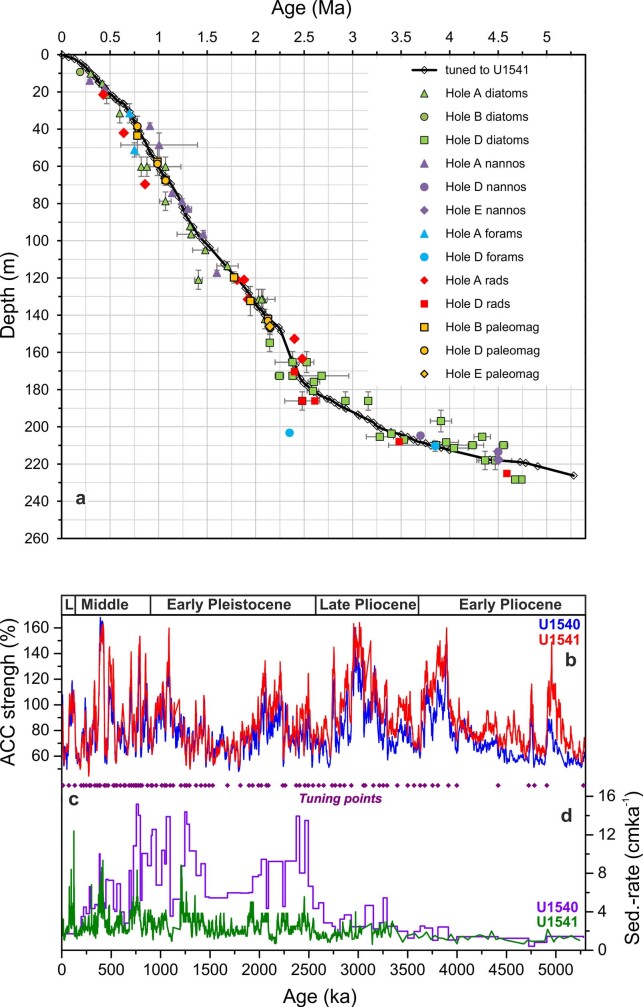
Stratigraphic background for IODP Site U1540. **a**, Age–depth plot for the Pliocene and Pleistocene sedimentary sequence at Site U1540 compared with biostratigraphic and palaeomagnetic tie points^24^. Vertical error bars represent the depth range of each stratigraphic marker (for example, the biostratigraphic ages were determined on core catchers during the expedition). Horizontal error bars represent age uncertainties adopted from the best-fit-interval output of the constrained optimization analysis of Southern Hemisphere biostratigraphic datasets. Palaeomagnetic ages are based on the geomagnetic timescale GTS2012. Further details are discussed in the methods section of ref. ^54^. **b**, ACC strength records of IODP Site U1540 tuned to Site U1541. **c**, Tuning points. **d**, Sedimentation-rate record at IODP Site U1540 and Site U1541.

To reconstruct the strength of the ACC and shifts of the frontal system over the past roughly 5.3 Myr, we use sediment records from the pelagic central South Pacific, the region farthest away from land in the global ocean (Fig. 1). Our study is primarily based on International Ocean Discovery Program (IODP) Expedition 383 Site U1540 and Site U1541, both drilled at about 3,600-m water depth within the Subantarctic Zone (SAZ, north of the Subantarctic Front (SAF))^24,25^ (Extended Data Fig. 1). IODP Site U1541 provides a continuous benthic foraminiferal stable oxygen-isotope stratigraphy back to around 3.5 Ma (ref. ^26^), with orbital tuning of sediment density to 41-kyr obliquity cycles between 3.5 and 5.3 Ma supported by shipboard biostratigraphic and palaeomagnetic time markers (Extended Data Figs. 2 and 3). The sedimentary record of IODP Site U1540 can be correlated to that of Site U1541 using X-ray fluorescence (XRF) core-scanner data (see Methods; Extended Data Fig. 4). To test the representativeness of ACC reconstructions at the IODP sites, we present further Pleistocene records along a meridional latitude transect (cores PS75/76, PS75/79 and PS75/83; Fig. 1).

We infer changes in ACC strength from sortable silt as proxy for near-bottom water-velocity variations^7,27^. Such records were previously used for reconstructing ACC strength changes at abyssal water depths in the vicinity of the Drake Passage^5,8^. Modern ACC studies suggest that eddy-field variations are important for short-term ACC variability and could compensate wind forcing completely when eddy saturation is reached^4^. However, averaging over centuries or more, the sortable-silt proxy represents a scalar mean water-column-integrated current speed^7,27^. Therefore, on longer timescales, the sortable-silt signal integrates the total water transport, including wind, baroclinic and eddy-induced contributions.

To reconstruct ACC strength, we infer sortable-silt records from high-resolution XRF core-scanner Zr and Rb data, calibrated with discrete grain-size measurements. Subsequently, we transfer the high-resolution records to absolute current strength using the sortable silt–flow speed correlation from the Scotia Sea^27^ (see Methods).

## Pleistocene ACC strength changes

**Fig. 2:**
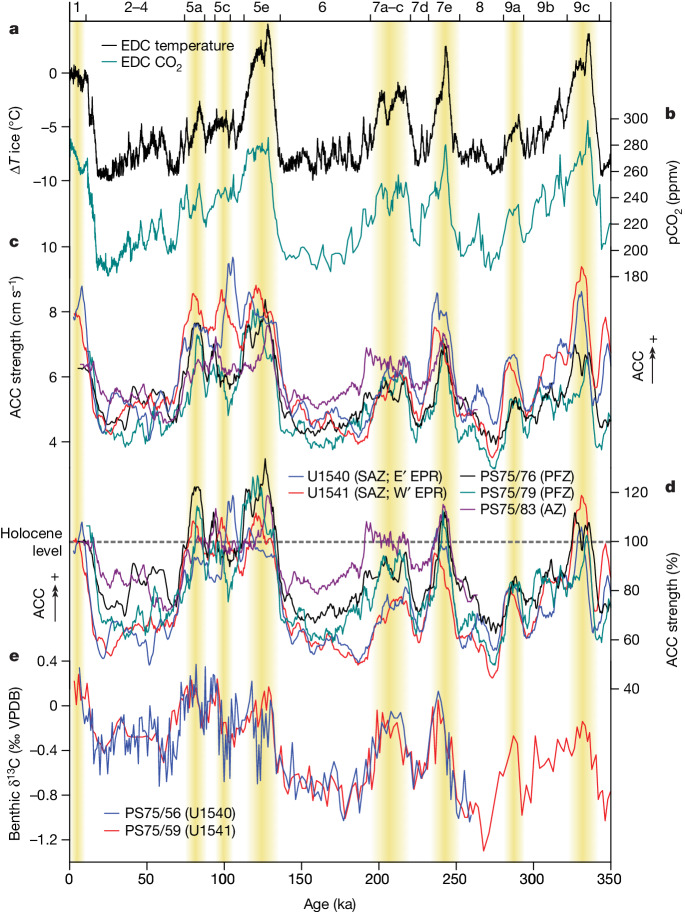
ACC strength changes over the past three glacial cycles (records along north–south transects from the SAZ to the AZ and west–east across the EPR in the SAZ) compared with Antarctic ice-core temperature and atmospheric CO_2_ records. **a**, Antarctic temperature record (EPICA Dome C (EDC) ice core)^52^. **b**, Atmospheric CO_2_ record (composite Antarctic ice cores)^53^. **c**, Reconstructed absolute ACC strength variations (cm s^−1^) from a cross-ACC transect, including the SAZ (Site U1540 and Site U1541), PFZ (cores PS75/76 and PS75/79) and AZ (core PS75/83) and across the EPR (eastern Site U1540 and western Site U1541). **d**, Reconstructed relative ACC strength variations (compared with Holocene mean values (dashed line)). **e**, Benthic foraminiferal δ^13^C records from core PS75/56 (same location as Site U1540) and core PS75/59 (same location as Site U1541). All sediment records were recovered from water depths bathed in Lower Circumpolar Deep Water masses at present. Numbers at top indicate MIS according to ref. ^10^.

Modern ACC flow between its northern and southern boundary fronts is not equally distributed across the Southern Ocean (Fig. 1). Most of the ACC transport occurs in the vicinity of the SAF, and less prominently at the northern boundary and the polar front (PF)^16^. To assess large-scale ACC strength changes and potential links to latitudinal shifts of the frontal system, we compare down-core records north–south across the ACC over the past three glacial cycles (0–350 ka) (Fig. 2). All records along the transect document similar absolute ACC strength (about 4–5 cm s^−1^) during glacial periods such as Marine Isotope Stage (MIS) 2–4 and 6, indicating homogeneously reduced glacial ACC flow across a broad latitudinal band. By contrast, during interglacials, we observe overall stronger and more variable ACC flow (about 6–9 cm s^−1^), with stronger flow in the SAZ compared with the Polar Frontal Zone (PFZ, between the SAF and the PF) (cores PS75/76 and PS75/79) (Fig. 2). Compared with the northern records, the Antarctic Zone (AZ) record (core PS75/83) shows lower-amplitude ACC changes with comparatively higher glacial values (about 5–6 cm s^−1^) and lower interglacial values (about 7 cm s^−1^) than the sites north of the PF (Fig. 2c). Relative to the Holocene mean, glacial ACC strength was reduced by about 30–50% in the SAZ, about 20–30% in the PFZ and at the PF and about 20% in the AZ, whereas ACC strength during interglacials MIS 5 and MIS 7 slightly exceeded the Holocene levels (Fig. 2d).

The largest decrease in glacial ACC flow occurred in the SAZ, the zone of strongest current transport under modern conditions^16^. Within the SAZ, we observe a similar magnitude of ACC strength reduction both to the west (Site U1541) and to the east (Site U1540) of the East Pacific Rise (EPR) (Fig. 1), excluding a strong effect of the topographic barrier of this mid-ocean ridge on ACC variability. This is also supported by consistently matching carbon isotope records from benthic foraminifera^28^ over the past three glacial cycles at these two locations (Fig. 2e). Therefore, we conclude that ACC strength records from Site U1540 and Site U1541, within the SAZ, are well suited to documenting the large-scale flow changes across the pelagic ACC in the Pacific Southern Ocean. Together, our records document a strong glacial ACC reduction spatially coherent across nearly the entire latitudinal range of the ACC in the central South Pacific during the past three glacial cycles. Conversely, during interglacials, we find an overall enhanced ACC that, at times, exceeded Holocene average flow, particularly in the SAZ.

**Fig. 3:**
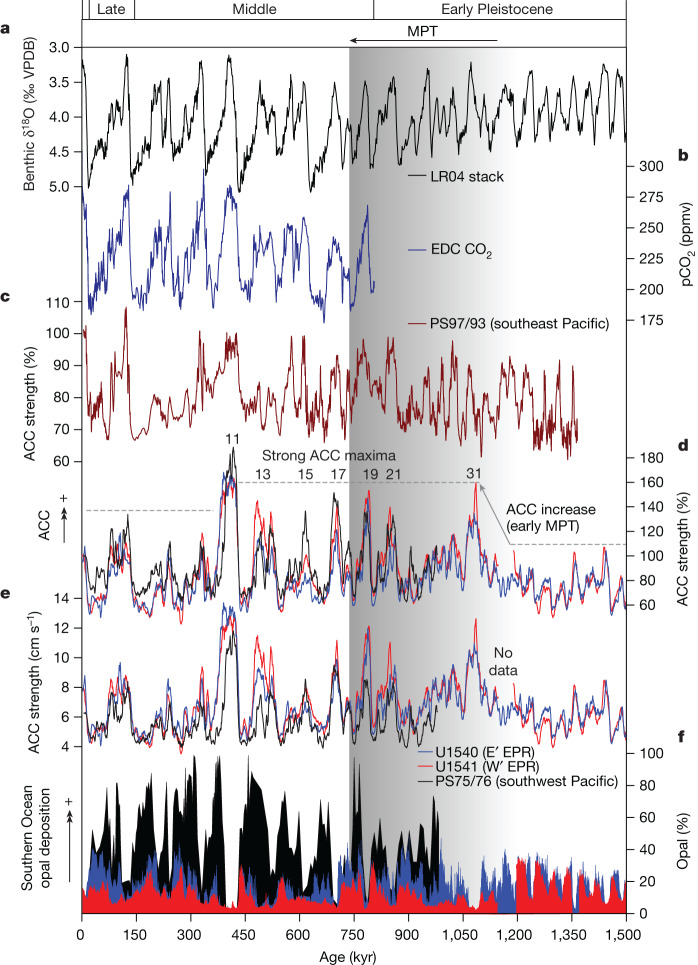
ACC development over the past 1,500 kyr. **a**, Benthic foraminifera oxygen isotope stack^10^. **b**, Atmospheric CO_2_ record (composite Antarctic ice cores)^53^. **c**, Relative ACC strength variations at core PS97/93, entrance of the Drake Passage^8^. **d**, Relative ACC strength variations at Site U1540, Site U1541 and core PS75/76. Dashed lines indicate different long-term interglacial ACC strength levels, with numbers marking MIS with outstanding interglacial ACC strength maxima. Black arrow indicates strengthening of the ACC during the early MPT. **e**, Absolute ACC strength variations at Site U1540, Site U1541 and core PS75/76. **f**, Opal-content changes at Site U1540, Site U1541 and core PS75/76.

Across the Middle and Late Pleistocene, our central South Pacific records document large-amplitude changes with strong ACC flow during interglacials between MIS 11 and MIS 21. Exceptionally strong ACC flow occurred during MIS 11 (150–180%), the highest values of the entire Plio-Pleistocene record, whereas ACC strength during interglacials MIS 13 to MIS 21 reached 130–150% of the Holocene ACC strength (Fig. 3). As for the three most recent glacial–interglacial cycles, glacials were characterized by reductions in ACC strength to similar levels at all sites, translating to roughly 50–70% of the Holocene estimates (Fig. 3). By comparison, the eastern South Pacific ACC strength record from the entrance of the Drake Passage (core PS97/93)^8^ revealed less pronounced glacial reductions (65–75%) and strongly attenuated interglacial maxima, with Holocene strength levels only slightly exceeded during relatively few warm intervals (Fig. 3c).

Pleistocene glacial–interglacial changes in opal content across our ACC transect document a clear opposite pattern in the SAF/PFZ compared with the AZ (south of the PF and north of the southern ACC front) (Fig. 3 and Extended Data Figs. 6–8), consistent with Atlantic Southern Ocean records^29^. These fluctuations are characterized by strongly increased opal contents across the SAF and the PF and reduced opal deposition in the AZ during glacials compared with interglacials. Ultimately, the opal records imply a relocation of Southern Ocean fronts that altered nutrient supply, stratification and iron fertilization in these surface ocean regions^29–32^. The glacial northward shift of the opal belt is accompanied by the overall homogeneous decrease of ACC strength across the entire latitudinal transect. During warmer Pleistocene interglacials, such as MIS 5, we observe a similar anticorrelation between opal deposition and ACC strength. Reduced interglacial opal deposition occurs in the SAZ, in which the strongest ACC flow is reconstructed. Conversely, enhanced interglacials opal deposition in the AZ occurs with only weak or modest enhancement of ACC flow compared with glacials, suggesting a clearer differentiation across the SAF and the PF (Fig. 2). Together, our ACC strength and opal-content records imply that both reduced overall current strength and latitudinal shifts of the fronts characterize glacial–interglacial Pleistocene ACC changes.

The MPT was a fundamental reorganization of Earth’s global climate system between about 1,250 and about 700 ka, when glacial–interglacial cycles changed from circa 41-kyr periods to circa 100-kyr periods and increased in amplitude^33^. Our ACC reconstructions exhibit a transition between around 1,300 and around 1,000 ka, with gradually increasing glacial and interglacial ACC strength coinciding with the early part of the MPT. This interval culminates in a pronounced ACC maximum during MIS 31, reaching about 160% of Holocene mean values. The increase in ACC flow strength in the SAZ during the initial part of the MPT is accompanied by the emergence of stronger orbital-scale fluctuations in opal contents at Sites U1540 and U1541 in the SAZ and in core PS75/76 located in the PFZ (Fig. 3). These fluctuations are characterized by strongly increased opal contents during glacials compared with interglacials, indicating a strengthening of the opal belts across the SAZ and the PFZ and/or a relocation of Southern Ocean fronts^26,29^.

## Long-term ACC development

**Fig. 4:**
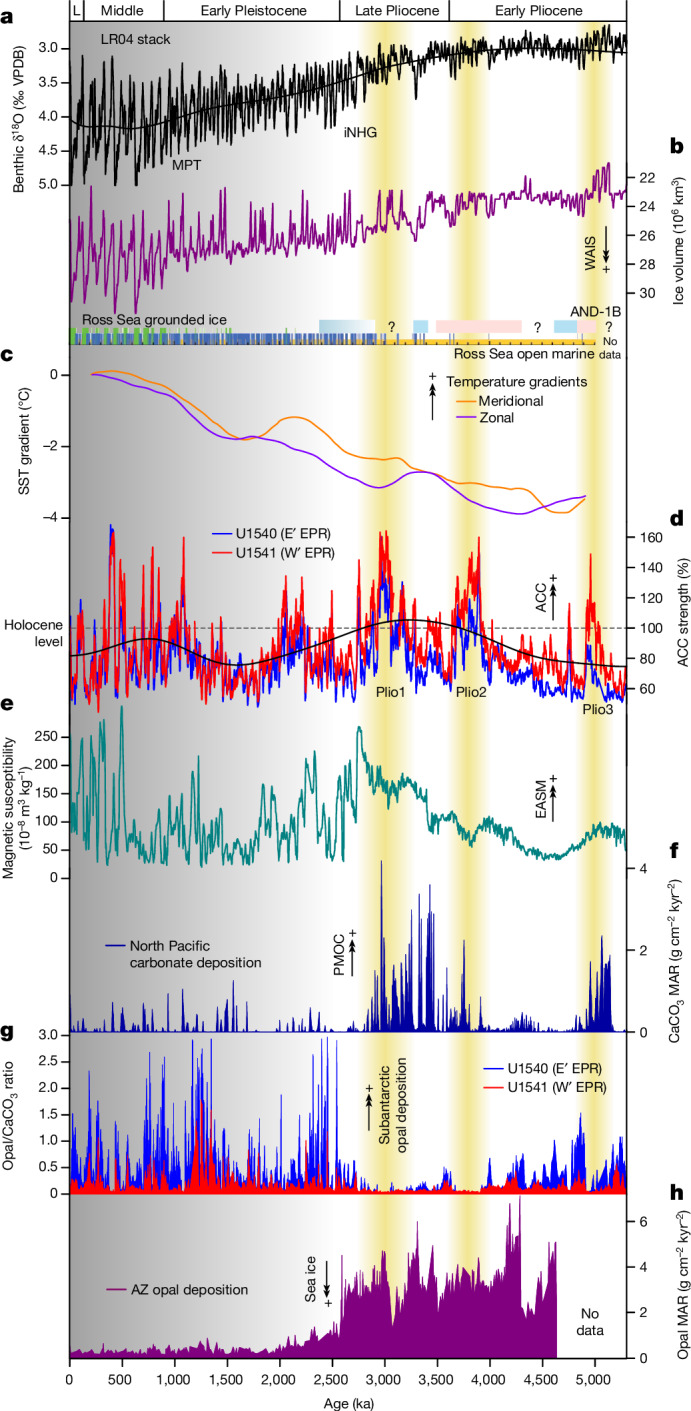
ACC development since the Pliocene. **a**, Benthic foraminifera oxygen isotope stack^10^. Black line shows the 1-million-year smoothed isotope record. **b**, Modelled Antarctic ice volume^46^ compared with the ANDRILL (AND-1B) ice-extent reconstruction (blue = advance; red = retreat; based on ref. ^12^), together with modelled sediment facies in the Ross Sea, close to AND-1B (yellow = open ocean; blue = floating ice; green = grounded ice)^1^. **c**, Pliocene to Pleistocene changes in meridional and zonal SST gradients. Negative values indicate gradient increase from the Pliocene to the Holocene^9^. **d**, Relative ACC strength variations (dashed line marks Holocene level) at Site U1540 and Site U1541. Black line shows the 1-million-year smoothed ACC strength record. Plio1, Plio2 and Plio3 mark long-term ACC maxima in the Pliocene. **e**, Magnetic susceptibility record from a loess–palaeosol sequence at the Chinese Loess Plateau^36^, indicating changes in the strength of the Asian monsoon. **f**, North Pacific record of carbonate mass accumulation rate (MAR) at Ocean Drilling Program (ODP) Site 882, indicating changes in the strength of the PMOC^38^. **g**, Changes in the ratio of biogenic opal to CaCO_3_ at Site U1540 and Site U1541. **h**, Changes in opal MAR at ODP Site 1096, indicating sea-ice extent and AZ ocean stratification^41^.

**Fig. 5:**
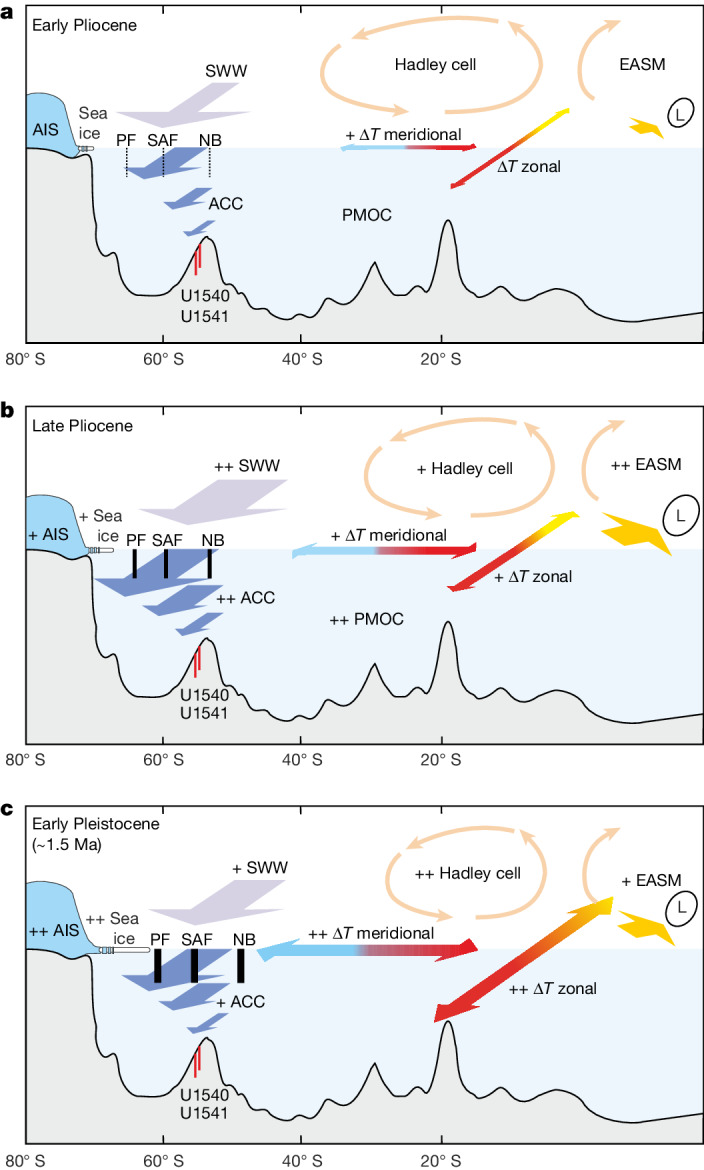
Schematic illustrating key atmospheric and oceanic processes influencing million-year trends in ACC strength. The schematics depict an idealized north–south transect from Antarctica across the Pacific (at about 125° W; north of 20° S out of scale). We illustrate the main atmosphere–ocean mechanisms influencing long-term changes in the ACC relative to the Early Pliocene. The Early Pliocene (**a**), the Late Pliocene before the iNHG (**b**) and the Early Pleistocene (1.5 Ma) situation following the Southern Ocean reconfiguration connected to the iNHG (**c**). U1540/U1541 = location of IODP sites; AIS, Antarctic ice sheet; NB, north boundary; Δ*T* = temperature gradients as in Fig. 4c; SWW, Southern Westerly Winds.

**Extended Data Fig. 5:**
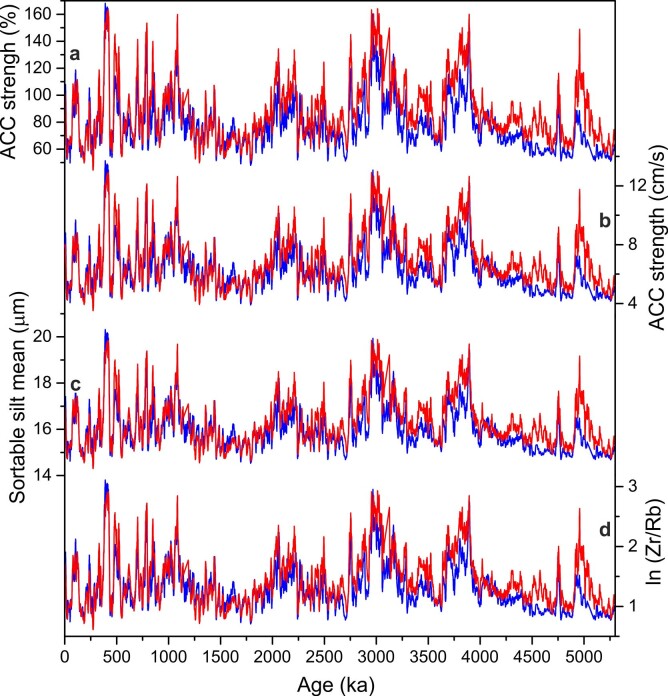
Raw data used for calculation of ACC strength at IODP Site U1540 and Site U1541. **a**, ACC strength records relative to the Holocene mean. **b**, Absolute ACC strength record calculated from sortable-silt data using a formula from the Scotia Sea in ref. ^27^ (see Methods). **c**, Sortable-silt record calculated from ln(Zr/Rb) using our calibration from discrete grain-size measurement (Extended Data Fig. 10; see Methods). **d**, ln(Zr/Rb) record (interpolated to 0.5 kyr and nine-point adjacent averaged).

Over the past 5.3 Myr, our sediment records document large variations in ACC strength, between roughly 50% and 180% of the mean Holocene ACC flow (around 3.5 cm s^−1^ to around 14 cm s^−1^ (Fig. 4 and Extended Data Fig. 5). Notably, we do not observe a linear, multimillion-year trend in ACC strength over the entire record, synchronous with the global cooling during this time period^9,10^. This is unexpected because, particularly in the Pacific Ocean, the multimillion-year cooling in global temperatures across the Plio-Pleistocene was accompanied by gradually increasing zonal and meridional SST gradients^9,23,34^. Taken at face value, increasing SST and atmospheric temperature gradients would strengthen the SWW and thus enforce the ACC^35^. Our ACC record documents this gradual increase in strength throughout the Pliocene (5.3–3.0 Ma; Fig. 4). However, after an ACC strength maximum in the Late Pliocene (about 3.0 Ma), ACC strength broadly declines, in opposition to expectations from continued Early Pleistocene cooling and ice-volume expansion (Fig. 4). These contrasting trends indicate that the ACC responded to fundamentally different forcings in the Pliocene versus the Early Pleistocene (Fig. 5). The shift in the ACC regime coincided with the marked climate reorganization associated with the intensification of the Northern Hemisphere glaciation (iNHG) that included global atmosphere–ocean circulation changes and increasing Antarctic ice-volume and sea-ice extent^11,13^.

During the Early Pliocene, the absence of a large marine-based Antarctic ice sheet strongly reduced sea-ice cover and weaker Southern Ocean density gradients^11,13^ would have resulted in weakly developed oceanic fronts (Fig. 5a). This setting would have enhanced the sensitivity of the ACC to atmospheric forcings, as oceanic forcings controlled by density gradients were plausibly weaker. The overall increasing trend in ACC strength during the Pliocene can thus be explained by overall increasing atmospheric forcing through the progressive equatorward movement and intensification of the SWW in response to decreasing global temperatures, increasing meridional temperature gradients and a progressive development of meridional Southern Ocean density gradients (Fig. 5a,b). The Pliocene changes parallel the beginning development of zonal gradients across the tropical Pacific^9^ and increasing East Asian Summer Monsoon (EASM) strength as recorded at the Chinese Loess Plateau^36^ (Fig. 4e). Proxy evidence for Pliocene EASM changes is heterogeneous across East Asia^37^ but modelling studies^37,38^ suggest that an expanded western Pacific warm pool and weakened zonal and meridional temperature gradients during the Early Pliocene reduced the EASM strength, superimposed on climatic consequences connected to the uplift of the Tibetan Plateau^36^. These changes in the Pliocene EASM, connected to large-scale zonal and meridional Pacific SST pattern, have a strong influence on tropical and subtropical atmospheric circulation, increasing the strength of both the Hadley and the Walker circulations. These changes plausibly enhanced the strength of the SWW and altered the latitudinal position of the SWW, including the high-altitude jet configuration (Fig. 5a,b).

In contrast to the Pliocene trend, we observe a weakening of ACC strength during the Early Pleistocene (until about 1.5 Ma; Fig. 4d). We propose that the processes driving meridional surface Southern Ocean density gradients during the Pleistocene were fundamentally different. During the Late Pliocene, global cooling associated with the iNHG and growth of Antarctic ice sheets would have cooled ocean temperatures in the AZ, intensifying the meridional temperature gradient until AZ waters reached the freezing point. Subsequently, further cooling would not have been possible in the AZ and instead cooling would have been concentrated north of the AZ. Thus, further Early Pleistocene cooling would instead decrease meridional temperature gradients in the mid-latitudes, the opposite sense as during the Pliocene (Fig. 5). A modelling study focusing on the effect of West Antarctic Ice Sheet (WAIS) growth across the iNHG simulates an increase of ACC strength^39^ in the Pacific sector, opposite to our proxy-based decreasing trend across this time period. This comparison suggests that either the advance of Antarctic ice sheets alone cannot explain the palaeo-ACC proxy records or likely relevant mechanisms and feedbacks are not represented in the climate model.

Superimposed on the Early Pleistocene enhanced high-latitude forcings, the decreasing ACC strength trend remains affected by zonal and meridional (sub)tropical SST gradients and the strength of the EASM (Fig. 4). In contrast to the Pliocene long-term trend, further increasing zonal temperature gradients across the tropical Pacific and overall decreasing EASM strength during the Early Pleistocene resulted in a decreasing long-term trend in ACC strength (Figs. 4 and 5c). These linkages are opposite to the Pliocene trends and strongly support our view of marked climate reorganization associated with the iNHG affecting the EASM^37^ and the southern high latitudes, including the ACC.

As well as ACC strength, the main changes across the iNHG are also evident in the biogenic sediment deposition at our sites (Fig. 4g). Whereas enhanced opal deposition occurs in the SAZ during intervals of reduced ACC strength throughout the Plio-Pleistocene, the opal content of SAZ sediments notably increases relative to carbonate at the iNHG. This shift in SAZ biogenic sediment deposition parallels coeval high-latitude changes, including increased opal burial in the Atlantic sector of the ACC^40^, decreased opal deposition in the AZ owing to increasing stratification and extended sea ice^13,41^, and notably decreased opal deposition in the subarctic North Pacific after about 2.75 Ma (refs. ^38,42^). These observations suggest that the Late Pliocene decrease in Pacific Meridional Overturning Circulation (PMOC), as indicated by stronger North Pacific carbonate deposition and/or preservation^34^ (Figs. 4f and 5b), led to a meridional redistribution of Pacific nutrient availability away from the North Pacific and AZ and towards the SAZ.

## Orbital forcing of ACC variability

On orbital timescales, the Plio-Pleistocene ACC strength records and changes in opal deposition are dominated by glacial–interglacial cycles and, notably, strong variations with an approximately 400-kyr period (Extended Data Fig. 9). These 400-kyr fluctuations of ACC strength are particularly strong during the Pliocene and Early Pleistocene, with large amplitudes of approximately 6 cm s^−1^ (Extended Data Fig. 5). Prominent intervals with above-modern (Holocene) ACC strength occur at around 2.8–3.1 Ma (Plio1), around 3.5–3.8 Ma (Plio2) and around 4.9–5.1 Ma (Plio3) (Fig. 4d). These Pliocene records are characterized by generally opposite variations in ACC strength and opal/carbonate ratios, with higher opal/carbonate ratios during times of reduced ACC strength (and vice versa; Fig. 4d,g). This pattern is consistent with the Pleistocene glacial–interglacial cycles and implies a strengthening and/or northward extension of the Pliocene opal belt during intervals with reduced ACC strength^29,30^, probably related to changes in upwelling of nutrients and ocean stratification. These changes are probably related to overall ACC strength changes and/or latitudinal shifts of the most likely weaker developed Pliocene ACC fronts (Fig. 5a).

The roughly 400-kyr cycles are evident in several Pliocene palaeoclimatic records, including marine oxygen isotope data and Asian monsoon records^36,43–45^, and are also present in simulations of Plio-Pleistocene Antarctic ice volume^46^ (Extended Data Fig. 9). They are thought to be an expression of long-term variations in the eccentricity of Earth’s orbit with the characteristic period of 400 kyr. A plausible mechanistic link to ACC changes could be through modulating atmospheric changes on precessional timescales^43^. For the past roughly 1 Myr, precessional forcing has been invoked to explain variations of the South Pacific jet stream related to the EASM and affecting the strength of the SWW and hence the flow strength of the ACC^14,47^. These model simulations and proxy results indicate a unique response of the jet-stream configuration in the SWW over the South Pacific to orbital forcing. During precession maxima, the split jet is strengthened, resulting in a reduced midlatitude jet and subantarctic SWW in the Pacific sector and thus reduced wind forcing of the ACC^14,47^. As for the Early Pleistocene million-year trend, the precessional changes are characterized by in-phase variations of zonal temperature gradients in the tropical Pacific and the EASM. By contrast, at the approximately 400-kyr band, the strengths of the EASM and the ACC are mostly antiphased (Extended Data Fig. 9). We suggest that EASM–ACC linkages might have operated differently because of the strong austral winter seasonal expression of the split jet changes^14,47^, its modulation by long-term eccentricity changes, as well as million-year timescale reconfigurations of low-latitude and high-latitude climate fluctuations affecting the ACC (Fig. 5).

A variety of palaeoproxy data point to a critical role of the Southern Ocean in influencing atmospheric CO_2_ content by affecting deep-water upwelling, the formation of new water masses and the Southern Ocean biological pump^2^. During the Middle and Late Pleistocene, glacial minima in ACC strength correspond to low global atmospheric CO_2_. This supports substantially reduced upwelling and stronger stratification, enhancing CO_2_ storage in the SAZ and PFZ, as previously shown for the last glacial cycle^48,49^. In contrast to the homogeneous decrease during glacials, enhanced ACC strength during individual interglacials was largely variable and not strictly linked to Antarctic temperature and the global atmospheric CO_2_ level (Fig. 3). Whereas continuous, orbitally resolved atmospheric CO_2_ reconstructions are not available for the Pliocene, we note a close covariance between maxima in marine carbon isotope (δ^13^C) records and eccentricity minima on the roughly 400-kyr timescale during the Pliocene and Early Pleistocene^45^ (Extended Data Fig. 9). The δ^13^C changes have been related to changes in the Southern Ocean carbon reservoir, involving deep and intermediate water stratification and marine productivity^45^. A connection (with changing phasing) of our reconstructed ACC strength changes to the approximately 400-kyr cycles in the global δ^13^C stack^50^ supports an important role for the ACC in shaping physical conditions for the marine carbon cycle, for time intervals before ice-core CO_2_ records.

## ACC strength and Antarctic ice sheets

ACC strength records are relevant for assessing the role of oceanic forcing for Antarctic ice-sheet development during the Pliocene. We observe that phases of ACC weakening paralleled advances of the WAIS as reconstructed from the Antarctic Drilling Project (ANDRILL)^1,12^, with ACC strengthening corresponding to WAIS retreat (Fig. 4). The first evidence for an advance of the WAIS in the Early Pliocene corresponds to an interval of reduced ACC strength following Plio3. Open marine conditions at the ANDRILL site (indicating WAIS retreat) occur after ACC maximum Plio2. A strong WAIS advance during the iNHG is paralleled by a decrease in ACC strength (Fig. 4). Moreover, approximately 400-kyr band-pass filters of ACC strength and modelled Antarctic ice-volume record^46^ are mostly antiphased over the Pliocene and Early Pleistocene (Extended Data Fig. 9), consistent with the expected relationship between a stronger ACC and ice-sheet retreat driven by enhanced southward advection and upwelling of Circumpolar Deep Water, together with southward-shifted oceanic fronts^1,12^. Conversely, Pleistocene interglacials (not covered by ANDRILL) with strong ACC circulation probably affected the stability of the WAIS. This comprises several super-interglacials during and after the MPT, notably including MIS 31 and MIS 11, which may have encompassed substantial WAIS retreat or even collapse^19^. Our reconstructions of strong ACC flow during these super-interglacials indicate that WAIS retreat or collapse may be mechanistically linked to substantially enhanced ACC flow. Our Plio-Pleistocene ACC reconstructions support the simulated roughly 400-kyr cyclicity of the Antarctic ice sheet with decreasing amplitudes after about 1.5 Ma. After MIS 31, strong glacial–interglacial cycles emerge and might be the consequence of dominating Northern-Hemisphere-paced climate cycles with the beginning of the MPT.

The ACC plays a crucial role in heat uptake and transfer to lower latitudes and ocean circulation on a global scale^17,18^. In this context, our palaeo reconstructions provide insights for global climate simulations that face substantial challenges in projecting future ACC and Southern Ocean changes and impacts on the carbon cycle^51^. Strong ACC flow, exceeding that of the preindustrial Holocene, mainly occurred during warmer-than-present time intervals during the Pliocene and Pleistocene interglacials. Observed ACC acceleration under anthropogenic warming (for example, intensified warming in the central South Pacific compared with the Drake Passage^17^) seem to match the patterns documented in our records of ACC strength maxima during interglacial warm intervals (Fig. 3c,d). These findings provide geological evidence in support of further increasing ACC flow with continued global warming. If true, a future increase in ACC flow with warming climate would mark a continuation of the pattern observed in instrumental records^17,18^, with probable negative consequences for the future Southern Ocean uptake of anthropogenic CO_2_.

## Methods

### Study locations

We analyse two Plio/Pleistocene sediment records recovered during IODP Expedition 383 (IODP Site U1540 and Site U1541)^54^ and three Quaternary records from piston cores obtained during RV Polarstern cruise ANT-XXVI/2.

IODP Site U1540 is located in the central South Pacific at 55° 08.467′ S, 114° 50.515′ W, about 1,600 nautical miles (nmi) west of the Magellan Strait at 3,580-m water depth^24^ (Extended Data Fig. 1). The site sits at the eastern flank of the southernmost EPR within the Eltanin Fracture Zone, roughly 130 nmi from the modern seafloor spreading axis, and is underlain by oceanic crust formed at the EPR about 6–8 Ma. The plate tectonic backtrack path of IODP Site U1540 moves the site westward, to an Early Pliocene position about 100 nmi closer to the crest of the EPR at a water depth shallower by several hundred metres. At a smaller scale, the site is located at the northeast end of a ridge that parallels the orientation of the EPR. IODP Site U1540 lies in the pathway of the subantarctic ACC, about 170 nmi north of the modern mean position of the SAF^55^. An approximately 213 m thick continuous sequence of Holocene to Early Pliocene sediments was recovered at IODP Site U1540. The sequence is dominated by carbonate-bearing to carbonate-rich diatom oozes, diatom-rich nannofossil and calcareous oozes.

IODP Site U1541 is located westward, at 54° 12.756′ S, 125° 25.540′ W, at 3,604-m water depth^25^ (Extended Data Fig. 1). The site sits on the western flank of the southernmost EPR, around 50 nmi north of the Eltanin-Tharp Fracture Zone and around 160 nmi from the modern seafloor spreading axis. IODP Site U1541 is underlain by oceanic crust formed at the EPR between about 6 and 8 Ma. As with IODP Site U1540, Site U1541 is located an Early Pliocene position roughly 100 nmi closer to the crest of the EPR. At a smaller scale, the site is located in a north-northeast–south-southwest-oriented trough, about 4 nmi wide, that parallels the orientation of the EPR. Site U1541 lies also below the pathway of the subantarctic ACC, about 100 nmi north of the modern mean position of the SAF^55^. An approximately 145-m spliced sedimentary sequence of Holocene–Miocene age was recovered at Site U1541. The sedimentary sequence includes four lithofacies: carbonate-bearing to carbonate-rich diatom ooze, diatom-bearing to diatom-rich nannofossil/calcareous ooze, nearly pure nannofossil ooze and clay-bearing to clayey biogenic ooze.

**Extended Data Fig. 6:**
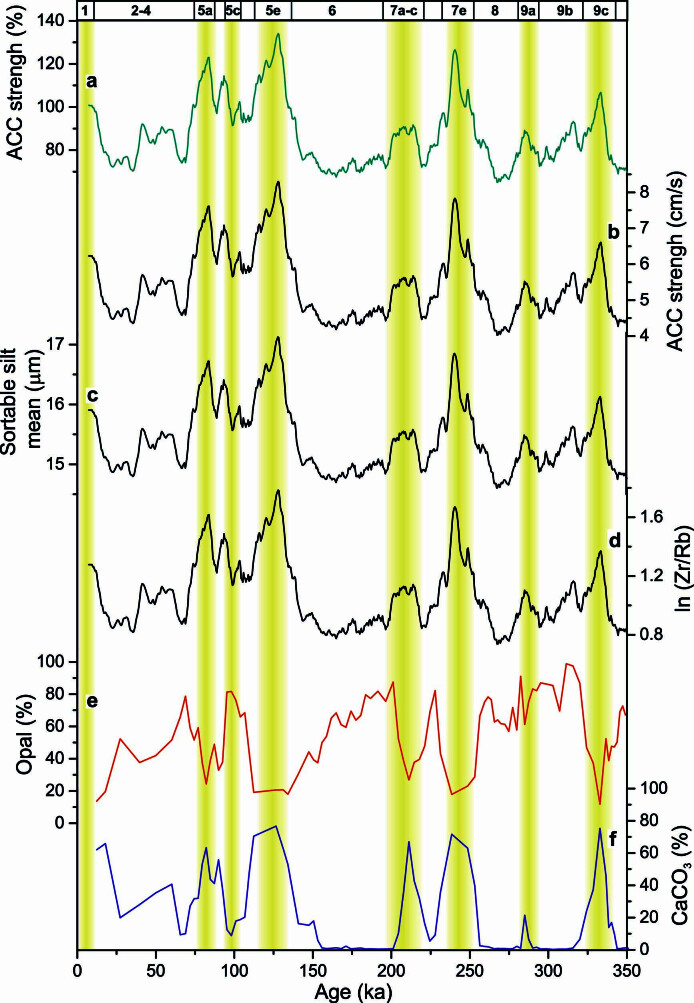
Raw data used for calculation of ACC strength together with opal and CaCO_3_ records from core PS75/76. **a**, ACC strength records relative to the Holocene mean. **b**, Absolute ACC strength record calculated from sortable-silt data using a formula from the Scotia Sea in ref. ^27^ (see Methods). **c**, Sortable-silt record calculated from ln(Zr/Rb) (Extended Data Fig. 10). **d**, ln(Zr/Rb) record (interpolated to 0.5 kyr and nine-point adjacent averaged). **e**, Opal content. **f**, CaCO_3_.

**Extended Data Fig. 7:**
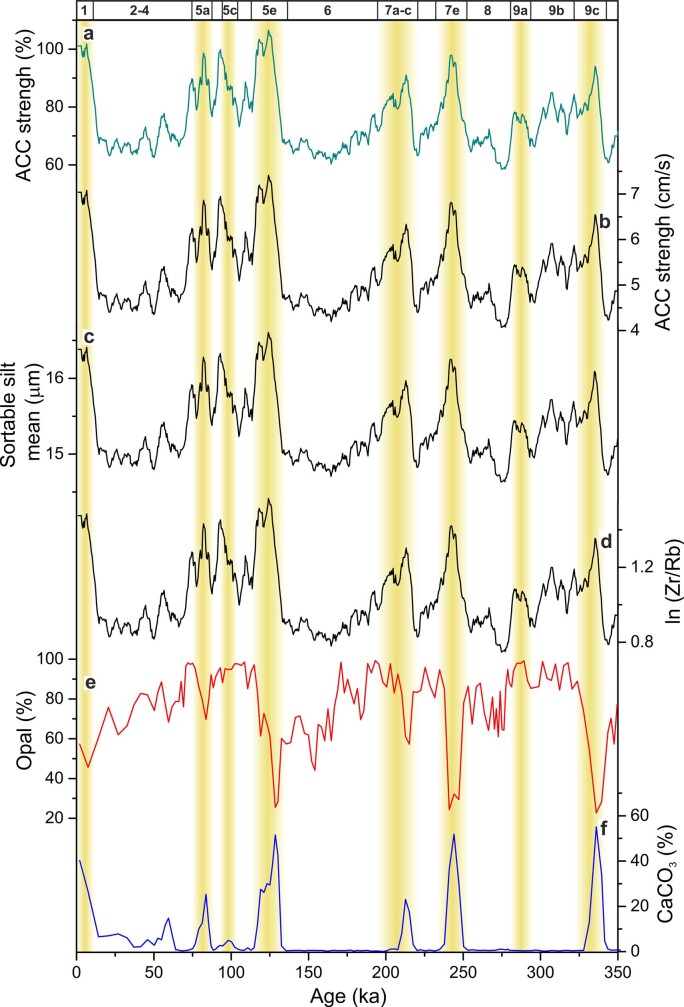
Raw data used for calculation of ACC strength together with opal and CaCO_3_ records from core PS75/79. **a**, ACC strength records relative to the Holocene mean. **b**, Absolute ACC strength record calculated from sortable-silt data using a formula from the Scotia Sea in ref. ^27^ (see Methods). **c**, Sortable-silt record calculated from ln(Zr/Rb) (Extended Data Fig. 10). **d**, ln(Zr/Rb) record (interpolated to 0.5 kyr and nine-point adjacent averaged). **e**, Opal content. **f**, CaCO_3_.

**Extended Data Fig. 8:**
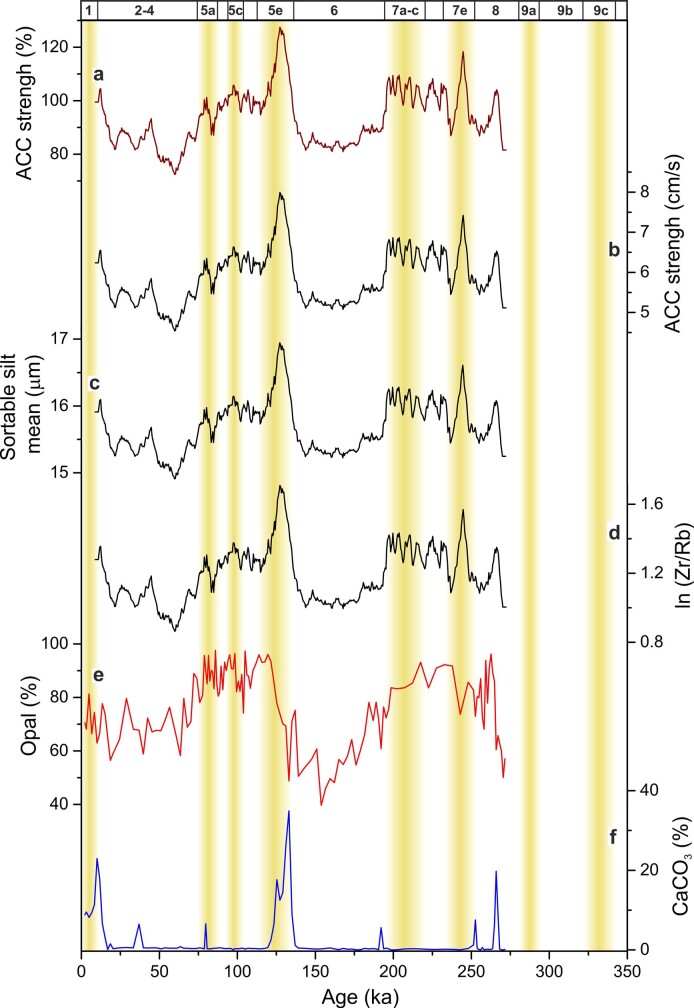
Raw data used for calculation of ACC strength together with opal and CaCO_3_ records from core PS75/83. **a**, ACC strength records relative to the Holocene mean. **b**, Absolute ACC strength record calculated from sortable-silt data using a formula from the Scotia Sea in ref. ^27^ (see Methods). **c**, Sortable-silt record calculated from ln(Zr/Rb) (Extended Data Fig. 10). **d**, ln(Zr/Rb) record (interpolated to 0.5 kyr and nine-point adjacent averaged). **e**, Opal content. **f**, CaCO_3_.

**Extended Data Fig. 9:**
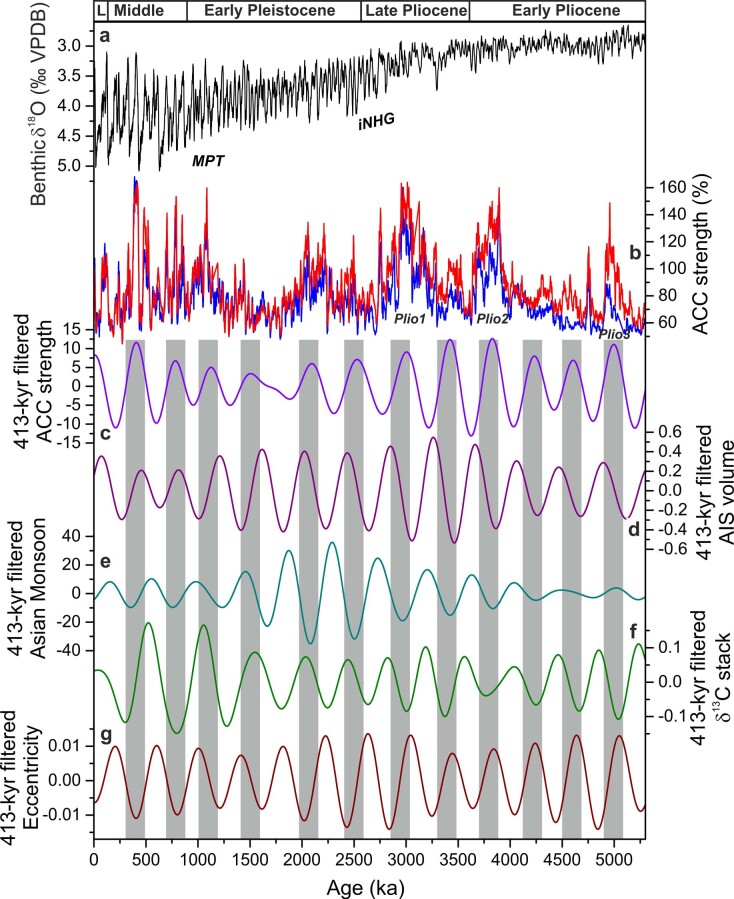
Long-term ACC changes on approximately 400-kyr timescales. **a**, Benthic foraminifera oxygen isotope stack LR04 (ref. ^10^). **b**, Relative ACC strength variations (dashed line marks Holocene level) at IODP Site U1540 and Site U1541. **c**, Filtered ACC record at Site U1541. Gaussian band-pass filter is centred at 413 kyr (0.00242 ± 0.0005 ka^−1^) as the main long-term eccentricity period^45^. **d**, Filtered volume record^46^ at 413 kyr. **e**, Filtered Asian Monsoon record^36^ at 413 kyr. **f**, Filtered global marine δ^13^C stack^50^ documenting global marine carbon reservoir changes. **g**, Filtered eccentricity parameter.

RV Polarstern cruise ANT-XXVI/2 cores include core PS75/76-2 (55° 31.71′ S, 156° 08.39′ W, 3,742-m water depth, core length 20.59 m) situated in the PFZ (Extended Data Figs. 1 and 6). Sediments are characterized by a cyclic succession of primarily calcareous oozes during interglacials and muddy siliceous oozes during glacials. Core PS75/79-2 (57° 30.16′ S, 157° 14.25′ W, 3,770-m water depth, length 18.51 m), located close to the modern PF, is dominated by siliceous oozes with carbonate restricted mainly to peak interglacials (Extended Data Figs. 1 and 7). Core PS75/83-1 (60° 16.13′ S, 159° 03.59′ W, 3,599-m water depth, length 13.13 m) was recovered from the AZ. Sediments are strongly dominated by siliceous oozes, with carbonate-bearing oozes appearing during interglacials (Extended Data Fig. 8).

### Age models

On the basis of the biostratigraphic and palaeomagnetic shipboard age-control points^54^, we further constrained the age model for Site U1541 from 0 to 3.4 Ma using the benthic foraminiferal oxygen isotope record and probabilistic tuning to Prob-stack^56^ (Extended Data Fig. 2). Middleton et al.^26^ use the hidden Markov model probabilistic algorithm (HMM-Match) from ref. ^57^ to align the U1541 benthic oxygen isotope data in three continuous segments with predefined start and end points of 0.00–31.35 m CCSF-A (0.000–1.126 Ma), 32.90–75.54 m CCSF-A (1.198–3.035 Ma) and 77.32–84.95 m CCSF-A (3.135–3.480 Ma), leading to two coring gaps between 31.78–32.75 and 75.67–77.12 m CCSF-A (ref. ^24^). The start and end points for each U1541 data segment were chosen through trial and error of visually determined alignment points that yielded the lowest uncertainties when run through the HMM-Match algorithm. From 3.4 to 5.3 Ma, we improved the shipboard record through orbital tuning of the GRA density record to obliquity (Extended Data Fig. 3).

The age model of IODP Site U1540 (Extended Data Fig. 4) is based on the biostratigraphic and palaeomagnetic shipboard age-control points^24^. We further improved the stratigraphy by correlating the ln(Zr/Rb) record to U1541 (Extended Data Fig. 4).

The age models of cores PS75/76, PS75/79 and PS75/83 were taken from ref. ^32^. We revised these age models, originally based on correlation of iron-content fluctuations to dust records from Antarctic ice cores, by using the non-continuous benthic foraminifera δ^18^O records available from these cores^32^.

### Stable oxygen and carbon isotope analyses on benthic foraminifera

Bulk sediments were freeze-dried and then washed with deionized water over a 150-µm mesh sieve to remove fine-grained material such as clay and silt. The coarse fractions of the sediment were subsequently dried in an oven at about 45 °C. From the coarse fraction larger than 150 µm, one to five specimens of the benthic foraminifera *Cibicidoides* spp. were picked with a wet brush under a stereomicroscope for stable oxygen and carbon isotope measurements. Samples were then analysed for stable oxygen and carbon isotopes (reported in δ notation with respect to the Vienna PeeDeeBee (VPDB) international standard, that is, δ^18^O and δ^13^C, respectively) at Lamont-Doherty Earth Observatory using a Thermo Delta V+ with Kiel IV. The NBS-19 international standard was analysed roughly every ten samples and the long-term one standard deviations for δ^18^O and δ^13^C of the NBS-19 standard are 0.06‰ and 0.04‰, respectively.

#### Geochemistry and bulk sediment parameters

Geochemical data were obtained through XRF scanning (at AWI, Germany and IODP, Texas A&M University, College Station, TX, USA) with an Avaatech (non-destructive) XRF core scanner. Split core surfaces were scanned at a 1-cm or 2-cm resolution during consecutive 10-kV, 30-kV and 50-kV runs, to obtain reliable intensities (area counts) of major elements and minor elements. We used the Zr and Rb intensities from the 30-kV run to calculate logarithmic ratios of both elements (ln(Zr/Rb)) used for the calculation of sortable silt and ACC strength (Extended Data Fig. 5).

We assess the strength and position of the ACC frontal system through reconstructing changes in the Southern Ocean opal belt, at present located in the PFZ (between the SAF and the PF)^30^. We use high-resolution physical properties data (density) and XRF-derived Ca counts calibrated by discrete biogenic opal and calcium carbonate content measurements (Methods).

For the determination of biogenic opal contents for sediment cores PS75/56, PS75/76, PS75/79 and PS75/83 and at Site U1541, we applied an automated leaching method at AWI, with a relative analytical precision of 2–5% (ref. ^58^). The high-resolution opal content records at Site U1540 and Site U1541 were obtained from polynomial regressions between GRA -density and the discrete biogenic opal measurements. At Site U1540, we used the regression from core PS75/56 from the same location.

For the SAZ records from Site U1540 and Site U1541, CaCO_3_ contents were used to calculate Opal/CaCO_3_ ratios. We used discrete CaCO_3_ content data from Site U1541 measured shipboard^24^ and data from core PS75/56 (ref. ^28^). At Site U1540, we used the calibration core PS75/56 from the same location. We obtained high-resolution carbonate records for U1540 and U1541 from XRF-based Sr count data calibrated with the discrete CaCO_3_ content measurements.

### Grain-size determinations and calculation of ACC flow strength

We infer changes in ACC bottom-water strength from grain-size estimates of fine-grained deep-sea and continental-margin sediments. Traditionally, this has been achieved by quantitative grain-size measurements of the terrigenous fraction using the mean grain size of sortable silt^27^ at continental margins and deep-ocean settings with bottom currents. More recent findings identified changes in element compositions of fine-grained sediments as a reliable proxy for the determination of grain sizes in the sortable-silt range that can be used to estimate bottom-current velocities^5,8,20,59^. Wu et al.^59^ showed that the logarithmic count ratio of zirconium to rubidium (ln(Zr/Rb)), as derived from high-resolution elemental records using XRF core-scanner data, is suitable to estimate bottom-current-speed changes. We apply the ln(Zr/Rb) proxy to calculate mean sortable-silt values and bottom-current speeds of the ACC back to around 5.3 Ma, using a regional calibration of discrete-sample sortable-silt measurements to XRF-scanner-derived ln(Zr/Rb) ratios (see below) and calculation of the current speeds following calibrations in ref. ^27^ (current speed = (sortable silt mean/0.59) – (12.23/0.59)) (Extended Data Figs. 5–8).

We use relative deviation from the Holocene mean current speed (except for the cross-frontal transect and Extended Data Figures showing also current speeds). The length, resolution and mean sortable-silt average across the individual Holocene sections vary among the records: U1540: around 0–10 ka, 8.14 µm; U1541: 0–6 ka, 7.9 µm; PS75/76: 0–11.5 ka, 6.18 µm; PS75/79: 0–11.5 ka, 6.93 µm; and PS75/83: 9–11.5 ka, 6.27 µm.

Grain-size distributions were obtained with a Beckman Coulter laser diffraction particle sizer LS13 320, equipped with a Micro Liquid Module at the Center for Marine Environmental Sciences (MARUM, University of Bremen, Germany). The lithogenic fraction was isolated from 300–500 mg of the bulk freeze-dried sediments by treating the samples with 5 ml H_2_O_2_ (37%), 5 ml HCL (10%) and 15 ml NaOH (20%) while being heated, to remove organics, carbonates and biogenic opals, respectively. The samples were rinsed and centrifuged until the pH was neutral between these steps. Directly before the measurements, a few drops of Na_4_P_2_O_7_·10H_2_O (sodium pyrophosphate) were added and the samples heated and sonicated to disaggregate the particles. Degassed water was used during analysis to minimise the effect of gas bubbles and a magnetic stirrer homogenised the sample during analysis. The resulting particle-size distributions range from 0.375 to 2,000 µm, divided into 92 size classes.

**Extended Data Fig. 10:**
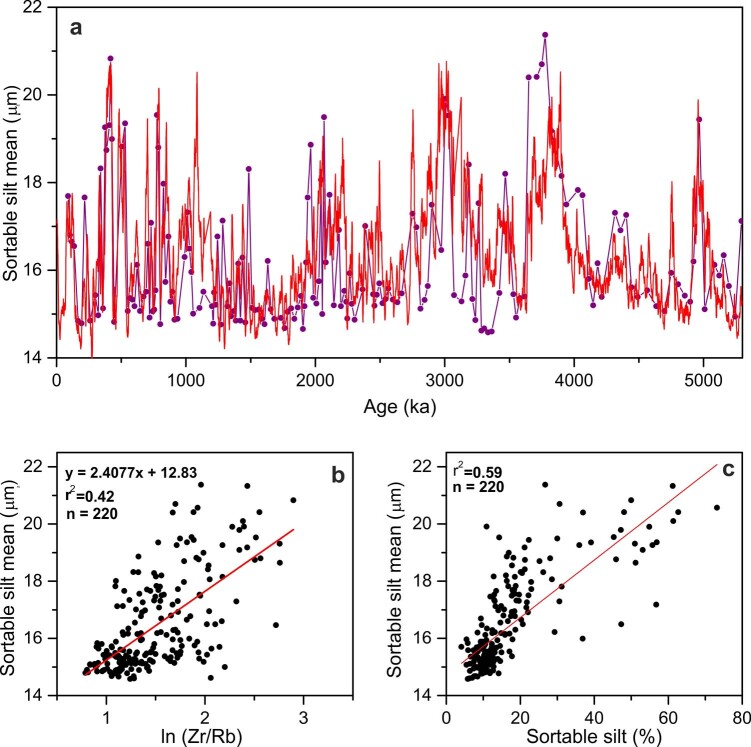
Raw data used for calculating mean sortable silt from ln(Zr/Rb) and correlation to sortable-silt percentages. **a**, Discrete sortable-silt mean measurements compared with the calculated record from ln(Zr/Rb) using the formula shown in **b**. **b**, Graphical correlation of sortable-silt mean values to ln(Zr/Rb). **c**, Positive correlation of sortable-silt mean and sortable silt percentage.

Sortable silt is defined as the mean grain size of the sortable-silt fraction (10–63 μm). We obtained a linear correlation between mean sortable silt and ln(Zr/Rb) ratios based on 220 samples at Site U1541 (sortable-silt mean = 2.4077 × ln(Zr/Rb) + 12.83) (Extended Data Fig. 10). The suitability of our sortable-silt data for bottom-current reconstructions is supported by the positive correlation of mean sortable silt and percentage of sortable silt (Extended Data Fig. 10). We excluded samples from MIS 11 with very high values that are outside the linear regression. We note that our positive linear correlation between ln(Zr/Rb) ratios and mean sortable silt has a lower slope compared with studies from the Southeast Pacific^8,20^. This might be explained by a different composition of siliciclastic material in the sortable-silt fraction at sites close to continental margins compared with our sites in the pelagic South Pacific.

We are aware that other factors, such as continental weathering, might affect the Zr/Rb ratio as a proxy for sortable silt and bottom-current speed. However, given the pelagic location of our sites, we conclude that, if a weathering influence would affect our central South Pacific records, this effect would be minor, given the large distance to any continent with substantial chemical weathering (in contrast, for example, to the Indian Ocean). Further support comes from above-mentioned records from the Southeast Pacific off Chile^20^ and the Drake Passage^5,8^, which provide excellent correlations of Zr/Rb to the mean sortable silt.

Although the standard analytical error of the grain-size analyses to obtain sortable-silt values are in the range ±0.6 µm (at 20 µm, see below), the exact error of the current-speed calculations from current-meter data is more difficult to assess, as only a few current-meter and grain-size data are available. McCave et al.^27^ estimated the standard error to be in the range ±12.5%.

## Online content

Any methods, additional references, Nature Portfolio reporting summaries, source data, extended data, supplementary information, acknowledgements, peer review information; details of author contributions and competing interests; and statements of data and code availability are available at 10.1038/s41586-024-07143-3.

### Supplementary information

Peer Review File

## Data Availability

All relevant data in this paper are available at PANGAEA Data Publisher (10.1594/PANGAEA.965443). Background images for Fig. 1 are from FESOM2 (Finite-volumE Sea ice–Ocean Model, formulated on unstructured mesh; https://fesom.de/). Extended Data Fig. 1 uses the Global Multi-Resolution Topography (GMRT) synthesis dataset as background data.
