# Supplementary material for: Five million years of Antarctic Circumpolar Current strength variability
Source: Nature. 2024 Mar 27;627(8005):789–96. doi: 10.1038/s41586-024-07143-3 (PMC10972744; doi:10.1038/s41586-024-07143-3)
Supplement: Supplementary file 1 — Peer Review File [file 41586_2024_7143_MOESM1_ESM.pdf]

Peer Review File

Manuscript Title: Five million years of Antarctic Circumpolar Current strength variability

Editorial Notes:

Reviewer Comments & Author Rebuttals

Reviewer Reports on the Initial Version:

Referees' comments:

Referee #1 (Remarks to the Author):

This study presents impressive new reconstructions of the strength of the Antarctic Circumpolar Current within the South Pacific sector over the last 5 million years. What is impressive about these sortable silt records is the degree of coherence in the inferred ACC strength changes across the sites along a North-South transect and across the East Pacific Rise. This data shows no pronounced long-term trend in ACC flow over the last 5-million-year despite the fact that there was a cooling of global climate and increase in global ice-volume over this time. Instead this data shows a link between ACC strength and 400kyr orbital eccentricity cycles.

Overall I think the novelty of these records makes this paper a strong candidate for publication in Nature. I do however have some concerns about the interpretation and narrative when it comes to inferring a relationship between these ACC strength records and other records, as discussed below.

Major comments:

Given that sortable silt is a proxy for bottom water velocity, a discussion is missing on the degree to which one should be concerned that changes in vertical density stratification over the time periods investigated can affect the amount of vertical shear within the ACC (Johnson and Bryden, 1989, Equation 7) and therefore how representative these reconstructions of bottom water velocities are of the column-integrated ACC strength.

The lack of a "long-term" trend in ACC strength.
The authors provide a reason for the 400,000k fluctuations involving the modulation of precessional changes in wind forcing, but they do not provide a clear explanation for why there is no long-term trend. The lack of a long-term trend in the strength of the ACC as climate cooled over the last 5Ma suggests that either there was no long-term trend in the strength/position of the westerlies (which would be surprising given the changes in large-scale SST gradients) or that there was a compensation between the changes in the westerlies and buoyancy forcing (Johnson and Bryden, 1989). Under the eddy saturation hypothesis (Hogg 2010) it is the meridional gradient in buoyancy forcing that exerts the primary control on ACC transport. The paper lacks a discussion of the possible role of changes in buoyancy forcing associated with changes in large-scale temperature gradients and sea-ice extent.

Lns 211-214 "Taken at face value, this would imply a long-term strengthening of the ACC, because increasing SST and atmospheric temperature gradients would strengthen the SWW driving the ACC. However, we do not observe such a long-term strengthening of the ACC over the Pliocene." Note that the long-term strengthening in the gradient in buoyancy forcing would similarly act to strengthen the ACC (e.g. see Figs. 1b, 3 & 4 in Hogg 2010), this implies that another mechanism is needed to

compensate. There could have been a progressive shift in the peak of the westerlies equatorward (e.g. Toggweiler et al., 2006) as climate cooled and the large-scale SST gradients strengthen. For example see Figure 9 in Burls and Fedorov (2014), there is a poleward shift of the westerlies in response to Early Pliocene warming patterns. Also note that in their Figure 7 there is a Pliocene shift in the peak poleward ocean heat transport at 60S.

Ln 246-248 – "The long-term modulation of these changes in the ~400-kyr cycle eccentricity band could therefore strongly overprint the expected increase in the SWW and ACC strength along the Plio-/Pleistocene global cooling." Perhaps this statement is trying to insinuate a role for shifts in the westerlies but is currently too vague. It is not clear how the imprinting of changes in the South Pacific Jet would act to remove the expected long-term trend.

The 400,000ka cycles in ACC strength. The authors find a "close link of ACC strength to the orbital eccentricity cycle with a period of ~400,000 years, identified in Antarctic ice volume simulations11, and likely originating from modulation of precessional changes in the South Pacific jet stream linked to tropical Pacific temperature changes12." Based on the climate model simulations of Lamy et. al. 2019, the hypothesis for weaker South Pacific Jet during precession maxima, decreasing southern westerly wind strength and hence the ACC seems plausible, but the authors need to provide more details on the mechanism, specifically the role tropical Pacific temperature changes mentioned in the abstract but not discussed in the main text.

The long-term changes in ACC strength and opal deposition are not as uniquely linked as the authors suggest. Lns 220-230 cites Cortese and Gersonde 2008 but misses the synthesis paper by Lawrence et al., 2013 which points to an equatorward shift in the westerly winds in both hemispheres to explain the productivity shifts (relevant to the westerly wind shift point above). Specifically that the high latitude Southern Ocean Site 1096 (see Fig. 1) displays similar behavior as the North Pacific in terms of the long-term change with Northern hemisphere glaciation. This point also applies to the discussion on Lns 261-263, Lawrence et al., 2013 discuss this from a global perspective.

Regarding the connection between 400kyr ACC changes and intermediate / deep-water formation in the subarctic North Pacific. Ln 252 states that "This phase of strongest PMOC as recorded in the opal deposition record from ODP Site 882" Note that while the opal record at 882 is interpreted by Burls et al. 2017 as a record for deep wintertime convection that reached down towards the depth of maximum silicate concentration (~1700 m in the modern North Pacific), the record for calcium carbonate accumulation rate is interpreted as a better measure of the strength of deep water formation, and by extension the PMOC, with high accumulation rates when the deep water formation ventilated all the way down to the depth of the sea floor at the site. These records are similar (Fig 1c vs 1d in Burls et al., 2017) but this should be kept in mine for the interpretation discussed on Lns 254-259.

Lns 282-289 – The mechanism behind the link between ACC strength and global delta13C stack needs to be fleshed out. Why and how they are linked is not clear in the current discussion.

ACC, ocean fronts, and AIS development. From Lns 293-295, it is not clear what one should be looking at in Fig 4 to support this statement. Perhaps I missed it but what is your proxy for AIS developments in Fig. 4? Is there a record connecting AIS advances with reduced southward advection and upwelling of the CDW, north-ward shifted fronts/SSW, and your records of ACC. If not, this part of the discussion feels very speculative.

The Mid-Pleistocene Transition and the glacial-interglacial coupling between ACC flow, the equatorial extent of the opal belt, and atmospheric CO2. Lns 193-195 – I don't see this change in the orbital-

scale fluctuations of opal content visually in 3f, please can you quantify this.

References cited

Burls, N. J., & Fedorov, A. V. (2014). Simulating Pliocene warmth and a permanent El Niño-like state: The role of cloud albedo. Paleoceanography, 29(10), 893-910.

Hogg, A. M. (2010). An Antarctic Circumpolar Current driven by surface buoyancy forcing. Geophysical Research Letters, 37(23).

Johnson, G. C., & Bryden, H. L. (1989). On the size of the Antarctic Circumpolar Current. Deep Sea Research Part A. Oceanographic Research Papers, 36(1), 39-53.

Lawrence, K. T., Sigman, D. M., Herbert, T. D., Riihimaki, C. A., Bolton, C. T., Martinez-Garcia, A., ... & Haug, G. H. (2013). Time-transgressive North Atlantic productivity changes upon Northern Hemisphere glaciation. Paleoceanography, 28(4), 740-751.

Toggweiler, J. R., Russell, J. L., & Carson, S. R. (2006). Midlatitude westerlies, atmospheric CO2, and climate change during the ice ages. Paleoceanography, 21(2).

Minor comments:

Title. What "emergence of coupled" meant in the title only made sense to me after reading the manuscript. I wonder if this can be reworded?

Abstract. Define "Long-term"

Abstract. "Antarctic ice volume simulations 11 and precessional changes in the South Pacific Jet linked to tropical Pacific temperature changes 12" -> the link to tropical Pacific temperature changes is not explicitly discussed in the discussion on Lns 239-248 so it is surprising to see this statement in the abstract.

Ln 95 "small variations in ACC strength documented" -> are documented

Lns 95-97 I found this sentence hard to follow

Ln 181 "Compared our" -> "Compared to our"

Ln 266-267 What do the authors mean by "diffuse deep PMOC"

Ln 370 e. -> f.

Referee #2 (Remarks to the Author):

A. This is a most interesting and potentially significant paper dealing with variations in the ACC flow strength in the central South Pacific. However it is not clear that the word 'Emergence of' in the title is warranted because significant variations in ACC strength have occurred since at least 5 Ma with a strong pulse at that point in time. And while there is a clear trend in global climate cooling since the Pliocene there is no such trend in the ACC flow strength, a point made in the opening paragraph. The

important point is that ACC strength does NOT match global cooling since the Pliocene.

B. Methods are not novel but the location is of the highest importance and the length of the record is far greater than any other.

C. This paper employs measurements of the sortable silt flow strength proxy and transfers them to the elemental ratio ln(Zr/Rb) for which there is are detailed records from XRF core scanning for the last 5.3 million years. The correlation on which this is based (extended data figure 9b) shows considerable scatter with r2 of only 0.42. A point that the authors will need to answer is that several of them, including Wu and Lamy, had a paper at the 2022 Bergen Paleoceanography conference dealing with the ACC in Drake passage which casts some doubt on the universality of the Zr/Rb relation to flow strength (Wu et al., 2022). Because in an earlier paper they had found coherent changes in sortable silt and Zr/Rb ratios, but in the work reported in the Bergen abstract the opposite was found, they said "variations of Zr/Rb ratio may thus have been primarily controlled by changes in weathering intensity". Clearly there are several controls of sediment compositional variations, of which flow speed control of grain size is one but climate-controlled weathering is another. Nevertheless, the relationship between sortable silt mean size and Zr/Rb ratio shown in extended data figure 9a is quite convincing. The authors refer to assessment of flow speeds following calibrations by McCave et al. (2017) but do not give the equation used (line 133 and extended data line 598). The point at issue here is that in February many users of the sortable silt proxy received a notification from McCave that the equations in a table in that paper were incorrect but those on the figure were good. It would be helpful to know whether Lamy et al. used the correct equation here.
The sortable silt measurements are plotted against age in extended data figure 9a but it is not stated from which core the samples were taken.

D. Uncertainies in transfer of ln(Zr/Rb) to grainsize and hence flow speed. not treated.

E/F. Lines 250 271, paragraph #14, contains the unresolved contrast between ACC and PMOC strength during Plio- 1 and the anti-phase Plio2 and 3 and continues with rather weak speculation concerning relationships between the North and South Pacific.

The next paragraph, (#15, 273-289), is also in the nature of a general discussion based mainly on previously published information. There are matters of importance in here; for example why does the ACC strength display a changing phase in relation to the carbon isotope stack, a point which is mentioned but not explained (see extended figure 8).
The next paragraph (#16, 291-310) relates important connections between fluctuations of the West Antarctic ice sheet (increases) to variations in ACC strength (decreases). However figure 4 referred to on line 299 does not contain any information regarding fluctuations in the WAIS. The remainder of the paragraph also contains several rather weak statements relating climate cycles, ice sheet cyclicity.
Paragraph #17 is a coda that draws attention to the likely consequence of a warming climate being an increase in ACC flow strength.
Overall the discussion paragraphs 14 to 17 (250-324) are rather more speculative and less tightly tied to the database presented here than would be expected in a paper for Nature, and more commonly associated with a journal such as Paleoceanography.
Line comments.
133. Check that the correct equation following McCave's correction notice has been used .
135-6. It would be helpful to clarify here that the density is a proxy for opal (because diatoms are hollow) and the calcium counts are a proxy for carbonate.
153. The ACC strength is frequently referred to as a percentage in figure captions; it would be helpful if, on first use in figure 2d, the percentage referred to is specified as the ratio of ln(Zr/Rb) to its Holocene mean (if my understanding is correct). Is Holocene here 0-11.7 ka?

164. Specify figure 2e. In several figure references later, particularly regarding figure 4, the letter designations of the panels are not given.
166. Why is the ACC here referred to as pelagic? Is this to distinguish it between the constrained ACC in the Drake passage, if so the point is obscure and the word could well be omitted.
181. ... Compared to our ...
211. After 3.8 Ma refer to figure 4f.
217 4c
223 4d
241. ....invoked not involved
263, 4d
346. Insert the meaning of percentage in ACC strength as suggested above.
370. e on this line should be f.
546. The stratigrapher was improved by ‘correlating’ not by ‘tuning’.
590-2. The point about the weathering control of the Zr/Rb ratio in Wu et al 2022 needs to be addressed here. It might also be worthwhile to include the definition of percentage strength as a percentage of the Holocene mean and specify what that value is and over what period it is averaged.
626. The red line in the upper part of the figure needs to be labelled STF and the caption needs to have subtropical front (STF) inserted. Because the fronts move about 5° further north during glacials the authors might say something about the relationship between U1541 and U1540 and the likely glacial positions of the fronts.
629 and 643. The fonts in these figure axis labels are too small.
663. Insert a ‘b’ for the second panel.
675. Insert a scale and label for the right-hand axis of panel a.
694, 697. Incorrect capitalisation.

G. see refs to references above.

H. From lines 250 to 324 could be tightened up and less speculative.

Referee #3 (Remarks to the Author):

Review of the manuscript "Emergence of coupled Antarctic Circumpolar Current flow strength and global climate cooling over the last 5 million years" by Lamy and co-authors

This manuscript uses (i) high-resolution XRF-based Zr/Rb ratios, calibrated with discrete sortable silt data; (ii) high-resolution biogenic opal contents, obtained from polynomial regression between GRA-density and discrete biogenic opal measurements; (iii) high-resolution CaCO3 contents obtained from XRF-based Sr count data, calibrated with the discrete CaCO3 content measurements and (iv) benthic foraminifera oxygen and carbon isotope data from two pelagic sediment cores in the Central South Pacific Ocean, IODP sites U1540 and U1541. Together with sites U1540 and U1541, three piston cores (PS75/76, PS75/79, PS75/83) were also studied in order to monitor ACC changes along a north-south transect in the Pacific sector of the Southern Ocean since the ~5.3 Ma.

The authors document a close link of ACC strength to the orbital eccentricity cycle with a period of 400,000 years and propose that this is likely originated from the modulation of precessional changes in the South Pacific jet stream linked to tropical Pacific temperature changes. Moreover, they suggest a stronger Pacific-wide circulation during the warm late Pliocene. They also report a link between weaker ACC flow, an equatorial extended opal belt and reduced CO_2 in glacial intervals first emerged during the Mid-Pleistocene Transition and persisted for the last 800 kyr. Lastly, they show that the strongest ACC flow occurred during warmer-than-present intervals of the Pliocene and Pleistocene.

Overall, the paper provides new important dataset for the past evolution of the ACC. Given the importance of the ACC for the global climate, these results are original and of major importance.

However, there are concerns on some of the interpretations and mechanisms proposed that I feel need to be addressed before publication. I have elaborated further below where I had key questions over the interpretation, but in summary I believe that more evidence is required to support some of the proposed mechanisms and conclusions.

I believe that the current title of the manuscript is not capturing the main message of the paper. I would suggest that the authors consider changing it.

Lines 92-100: Given that several records along the Southern Ocean point to regional heterogeneity of ACC flow in the different sectors of the Southern Ocean over Pleistocene glacial-interglacial cycles, and your records do not show a long-term trend in ACC flow, following the gradual global cooling and increase in global ice-volume, I am not convinced (however do not completely disagree) that a north-south transect across ACC in the Pacific sector of the Southern Ocean could really provide insights on the overall ACC fluctuations. Is it possible that you record a more regional view of the dynamics of the ACC?

As you mention in lines 105-108: reconstructions of southern hemisphere meridional SST gradients indicate an overall strengthening of the atmospheric circulation (SWW) over the Pliocene and early Pleistocene, that would most plausibly imply an enhancement of the ACC. So, why you do not see an overall long-term increase in ACC flow speeds in the Central Pacific? I think this is an interesting finding that needs further explanation.

Line 108: You could consider adding a more recent reference - SST record offshore Tasmania (IODP Site 1168) (Hou et al. 2023; https://doi.org/10.5194/cp-19-787-2023)

Line 110: I would delete the word “position”. Although you document shifts of the ACC frontal system, you do not reconstruct the position of the ACC during the study interval.

Lines 123: It would be very useful to show the depths of the piston cores too, as well as provide references associated to this information.

Line 153. You should mention which cores and the mean SS values you used to calculate the mean Holocene flow value (maybe add it in methods).

Lines 170-171: “The spatial coherence of glacial decrease does not necessarily require large latitudinal 171 shifts of the ACC and SWW”. Again, in lines 198-200, you suggest that the changes you observe in your opal % records do not necessarily require a substantial latitudinal shift of the opal belt. As you do not provide any data, this is rather speculative. How can you differentiate from the low/high opal % shown in your data if they are controlled by lower/higher biogenic productivity or/and latitudinal migration of the frontal system and thus shifts of the opal belt? Although not shown in the manuscript or in the supplementary data, I understand that you have opal and XRF Ca data from all sites (IODP and PS cores). Since you have a north-south transect across the ACC frontal system, you could use them, together with diatom/calcareous counts (if available) to check if there were any substantial latitudinal migration. Good/bad preservation of microfossils can also affect your results. SEM images on siliceous and calcareous microfossils will help to strengthen your arguments.

Saying that, I am a little confused when in lines 220-225: you suggest that the opposite variations between ACC strength and opal/carbonate ratios implies a northward extension of opal belt during glacials with reduced ACC strength. I think you need to provide stronger evidence on your interpretation on the opal % and opal/carbonate ratios.

Lines 181-185: Why there is such as difference in the mean SS values between your sites and PS97/93 given that they are all at a similar depth?

Lines 205-214. As I mentioned before, this is a very interesting observation that I believe needs more explanation. Why you do not see any long-term strengthening of the ACC following the global cooling and intensification of SWW? Does this suggest a local response rather than a more general ACC response?

Lines 259-271: I think that this section needs to be developed a bit more and specific connections are to be made between your data and available records. In this current version it is very speculative.

Lines 294-300: This part was hard to follow as you cite figure 4 for the comparison between the WAIS retreats /advances and the variations in the ACC strength, but you do not show any data for the WAIS dynamics. Since you propose an important link between the ACC, frontal system and Antarctic ice sheet, I believe you should add available published data reporting WAIS dynamics in Fig.4.

Main Figures:

There is a mistake in the colors between the legends and the data lines of the piston cores plotted in Figure 2c, 2d.

Line 370, Figure 4: Replace “e” with “f “in the figure caption

Methods:

Line 552: How many samples in total were analyzed?

Mean sortable silt and Ln(Zr/Rb) raw data from the three piston cores used for calculation of ACC strengths at these sites are missing. A figure similar to Extended Data Fig. 7 is needed in the supplementary material. Similarly, you should add an additional figure in the supplementary material showing the XRF- Ca counts, discrete biogenic opal and calcium content measurements for the studied cores.

Line 676: The correlation between mean SS and Ln(Zr/Rb) shown in Extended Fig Data 9b is rather weak (0.42), compared to Wu et al., 2020 (0.83). In line 619-621: You mention that this might be explained by a different composition of siliciclastic material in the SS fraction. Could you provide more details?

Extended Data Fig. 9a: Add units and values of ln(Zr/Rb) in the right side of the figure.

Author Rebuttals to Initial Comments:

Response to Referee #1 (Pliocene climate evolution; PMOC):

(comments by the reviewer are printed in italics, our point-by-point response in blue)

This study presents impressive new reconstructions of the strength of the Antarctic Circumpolar Current within the South Pacific sector over the last 5 million years. What is impressive about these sortable silt records is the degree of coherence in the inferred ACC strength changes across the sites along a North-South transect and across the East Pacific Rise. This data shows no pronounced long-term trend in ACC flow over the last 5-million-year despite the fact that there was a cooling of global climate and increase in global ice-volume over this time. Instead this data shows a link between ACC strength and 400kyr orbital eccentricity cycles.

Overall, I think the novelty of these records makes this paper a strong candidate for publication in Nature. I do however have some concerns about the interpretation and narrative when it comes to inferring a relationship between these ACC strength records and other records, as discussed below.

We thank Referee #1 for the very positive assessment of our paper.

Major comments:

Given that sortable silt is a proxy for bottom water velocity, a discussion is missing on the degree to which one should be concerned that changes in vertical density stratification over the time periods investigated can affect the amount of vertical shear within the ACC (Johnson and Bryden, 1989, Equation 7) and therefore how representative these reconstructions of bottom water velocities are of the column-integrated ACC strength.

We thank the Reviewer for this valuable comment. We agree that variations in the vertical density structure might critically affect bottom water velocities. Most oceanographic information on the vertical flow across the ACC originates from "choke points" of the circumpolar flow, i.e., primarily Drake Passage and, more recently, from the central Pacific Udintsev Fracture Zone (UFZ; west of our IODP coring sites at the East Pacific Rise and east of our core transect at the Pacific Antarctic Ridge). In the Drake Passage ACC transport below 3000 m is relatively minor, mostly due to the influence of deep local circulation cells (Koenig et al., 2014). At the UFZ, an ACC transect shows deep-reaching flow in vicinity of the fracture

zone (bathymetrically enhanced) and at the northern subantarctic front, where another fracture zone is located (Park et al., 2019).

Based on the water depth (> 3500m) and location of our sites away from fracture zones, we expect that the ACC at our sites is on average less affected by bottom topography and the location of fracture zone. Key support for the robustness of our reconstruction comes from the meridional transect of sites from the subantarctic to the Antarctic zone in the central South Pacific across a large range of varying sedimentary regimes and conditions (Fig. 2). The overall consistent glacial/interglacial variations along the transect and the consistent Plio/Pleistocene long-term changes in ACC strength at our sites East and West of the EPR suggest that more local vertical density structure changes probably did not a play a major role on the time scales of interest.

The lack of a "long-term" trend in ACC strength.

The authors provide a reason for the 400,000k fluctuations involving the modulation of precessional changes in wind forcing, but they do not provide a clear explanation for why there is no long-term trend. The lack of a long-term trend in the strength of the ACC as climate cooled over the last 5Ma suggests that either there was no long-term trend in the strength/position of the westerlies (which would be surprising given the changes in large-scale SST gradients) or that there was a compensation between the changes in the westerlies and buoyancy forcing (Johnson and Bryden, 1989). Under the eddy saturation hypothesis (Hogg 2010) it is the meridional gradient in buoyancy forcing that exerts the primary control on ACC transport. The paper lacks a discussion of the possible role of changes In buoyancy forcing associated with changes in large-scale temperature gradients and sea-ice extent.

Please refer to our detailed response below the next paragraph.

Lns 211-214 "Taken at face value, this would imply a long-term strengthening of the ACC, because increasing SST and atmospheric temperature gradients would strengthen the SWW driving the ACC. However, we do not observe such a long-term strengthening of the ACC over the Pliocene." Note that the long-term strengthening in the gradient in buoyancy forcing would similarly act to strengthen the ACC (e.g. see Figs. 1b, 3 & 4 in Hogg 2010), this implies that another mechanism is needed to compensate. There could have been a progressive shift in the peak of the westerlies equatorward (e.g. Toggweiler et al., 2006) as climate cooled and the large-scale SST gradients strengthen. For example see Figure 9 in Burls and Fedorov (2014), there is a poleward shift of the westerlies in response to Early Pliocene warming patterns. Also note that in their Figure 7 there is a Pliocene shift in the peak poleward ocean heat transport at 60S.

We thank Referee #1 for critically examining the long-term Plio-/Pleistocene trend in our records and potential forcing mechanisms. These very relevant comments mirror similar points raised by Reviewer #3.

In the original manuscript, we referred to the absence of a linear "long-term trend" across the entire time-series (i.e., 5.3 Ma), in contrast to the linear trends in global climate (e.g., the Lisiecki/Raymo $\delta^{18}O$ stack and Pacific SST records) over this time period. Along with Referee #3, Referee #1 pointed out that the overall Plio-Pleistocene climate evolution from warmer-than-present early Pliocene conditions (with reduced meridional SST gradients and small Antarctic ice-sheets/sea-ice extent) to the Pleistocene bi-polar glaciation (with enhanced ocean-atmospheric gradients) implies strengthening of the westerly winds together with Southern Ocean density gradients, both of which would enhance the ACC. This overall configuration is challenging to reconcile with the missing long-term linear trend in our ACC strength time-series.

Inspired by the Referee's comment, we re-assessed the "long-term" trends in light of other feasible forcing mechanisms for ACC strength changes over the past 5.3 Ma. Whereas there is no linear long-term trend over the entire length of the record (as stated in our original manuscript), there are trends beyond the ~400 kyr cycles. The one million-year-smoothed ACC strength record reveals a long-term increasing trend throughout the Pliocene cooling trend (Fig. 4). In contrast to the Pliocene, during the early Pleistocene (until ~1.5 Ma), decreasing ACC strength parallels global climate cooling and ice volume increase. Thereafter, ACC flow increases again towards the Mid-Pleistocene-Transition (MPT).

In order to explain these contrasting trends, we propose that the ACC responded to fundamentally different forcings during the Pliocene compared to the Pleistocene. The shift in the ACC regime occurred with the major climate reorganisation connected to the intensification of the Northern Hemisphere Glaciation (iNHG) that was accompanied by major atmosphere-ocean circulation changes and increasing Antarctic ice-sheets and sea-ice extent.

During the Pliocene, the absence of a major marine-based Antarctic ice-sheet, the strongly reduced sea-ice cover, and weakened density gradients across the Southern Ocean compared to the Pleistocene, led to less developed oceanic fronts. Such a Pliocene Southern Ocean setting would have strengthened the sensitivity of the ACC to atmospheric forcings compared to oceanic forcings. The Pliocene increasing trend in ACC strength can be plausibly explained by overall increasing atmospheric forcing, consistent with a progressive equatorward movement and intensification of the westerlies in response to decreasing global temperatures, increasing latitudinal temperature gradients, and growing development of Southern Ocean density gradients. The Pliocene atmosphere-ocean changes also parallel the initiation of zonal gradients across the Eastern Pacific cold tongue and increasing Asian monsoon from ~4.2 to 2.75 Ma (Fig. 4). Together, these changes plausibly contributed to enhanced wind forcing of the Pacific ACC through teleconnections

involving the southern Hadley cells and the southern westerlies. It is conceivable that Pliocene tectonic changes, such as uplift of the Tibetan Plateau affected gradual trends in the Asian monsoon (Nie et al., 2014; Sci Rep.). However, models do not suggest a major impact of the final closure of the Central American Seaway on large-scale Pacific circulation, including ACC strength (e.g. Burls et al., 2017, Sci. Adv.).

The shift from the Pliocene to the Pleistocene ACC response occurred after the late Pliocene ACC maximum *Plio 1* with a major decrease of ACC strength. This change is supposedly connected to the major global climate shift involving the iNHG, and a further step in the development of Antarctic ice-sheets, particularly the West Antarctic Ice Sheet (WAIS) (McKay et al., 2012; PNAS). We suggest that major changes around Antarctica, e.g., sea-ice extent and deep-water formation, strengthened the latitudinal ocean density gradients, thereby enhancing Southern Ocean fronts and displacing them northward. This scenario in all likelihood explains the concomitant increase in opal deposition at our sites and the decrease in the Antarctic Zone (e.g., at ODP Site 1096) (now plotted in Fig. 4k).

In the revised manuscript (lines 209 to 221), we now discuss that this fundamental reconfiguration of the Southern Ocean altered the sensitivity of the ACC to the different atmospheric and oceanic forcings. In contrast to the Pliocene, during the early Pleistocene (until ~1.5 Ma), global climate cooling and ice volume increase is paralleled by a multi-million-year trend of decreasing ACC strength. This is in contrast to the expected increasing ACC strength due to stronger atmospheric and oceanic gradients. As pointed out by the referees concerning the long-term trend along the entire record, the early Pleistocene pattern of opposite changes in global temperature/ice volume and ACC strength requires compensating mechanisms resulting in a net decrease of ACC strength.

We propose two major factors:

(a) Changes in ocean density gradients: We propose that after global cooling along with the intensification of the NHG and the build-up of Antarctic ice-sheets, ocean temperatures in the Antarctic Zone approached the freezing point and thus further Southern Ocean cooling decreased meridional temperature gradients in the southern mid-latitudes (and did not not enhance these gradients as would have occurred during the Pliocene). Thus, latitudinal density gradients became weaker across the Southern Ocean fronts with progressive cooling. The same mechanism ostensibly contributes to glacial-interglacial changes with the weaker ACC during glacials (e.g., Lamy et al., 2019; PNAS).

(b) Changes in atmospheric forcings related to the South Pacific Split Jet. In our original manuscript, we discuss the ~400-kyr cycles in terms of long-term changes in the configuration the Pacific branches of high-altitude atmospheric jets affecting the wind forcing of the ACC. In this concept, a reduced split jet enhances ACC wind stress forcing as shown by modelling studies (e.g., Chiang et al., 2018; Lamy et al., 2019). Changes of the split jet are linked to zonal SST gradients in the tropical Pacific and the strength of the Asian monsoon. Decreased zonal gradients and stronger monsoon weaken the split jet and strengthen the subantarctic westerlies in the Pacific sector, and thus the ACC. This mechanism is also consistent with the long-term Pliocene enhancement of the monsoon and the ACC.

After the long-term ACC strength minimum at ~1.5 Ma, the one million-year-smoothed ACC strength record increases again, coinciding with a major SST cooling in the subarctic and subantarctic zones (Martinez-Garcia et al., 2010). This cooling and connected strengthening of atmospheric circulation substantially increased dust supply to the South Atlantic towards the MPT (Martinez-Garcia et al., 2011). These patterns are consistent with the strong glacial-interglacial amplitudes of the ACC strength with the beginning of the MPT at ~1.3 and again at ~1 Ma.

We have added a respective part to the main text describing the trends and proposed mechanistic links in section "Long-term ACC development", lines 209 to 260.

Ln 246-248 – "The long-term modulation of these changes in the ~400-kyr cycle eccentricity band could therefore strongly overprint the expected increase in the SWW and ACC strength along the Plio-/Pleistocene global cooling." Perhaps this statement is trying to insinuate a role for shifts in the westerlies but is currently too vague. It is not clear how the imprinting of changes in the South Pacific Jet would act to remove the expected long-term trend.

The reviewer is correct in suggesting that this more general statement invoked latitudinal shifts of the westerlies. We agree that the prior statement was too vague and we have extended the general discussion of the long-term trends including the role of latitudinal shifts of the westerlies as outlined above.

The 400,000ka cycles in ACC strength. The authors find a "close link of ACC strength to the orbital eccentricity cycle with a period of ~400,000 years, identified in Antarctic ice volume simulations11, and likely originating from modulation of precessional changes in the South Pacific jet stream linked to tropical Pacific temperature changes12." Based on the climate model simulations of Lamy et. Al. 2019, the hypothesis for weaker South Pacific Jet during precession maxima, decreasing southern westerly wind strength and hence the ACC seems plausible, but the authors need to provide more details on the

mechanism, specifically the role tropical Pacific temperature changes mentioned in the abstract but not discussed in the main text.

On precessional time-scales, Lamy et al. (2019; PNAS) found a stronger split of the South Pacific jet during precession maxima linked to enhanced zonal SST gradients in the tropical Pacific. During a stronger split jet, the mid-latitude jet and overall southern westerly winds are weaker and decrease the atmospheric forcing of the ACC. This is also seen in the modelling study by Chiang et al. (2018; Paleoceanography), explicitly modelling reduced Drake Passage throughflow with a stronger split jet. We expanded our explanation of the split jet in the main text accordingly (line numbers 280-290).

The long-term changes in ACC strength and opal deposition are not as uniquely linked as the authors suggest. Lns 220-230 cites Cortese and Gersonde 2008 but misses the synthesis paper by Lawrence et al., 2013 which points to an equatorward shift in the westerly winds in both hemispheres to explain the productivity shifts (relevant to the westerly wind shift point above). Specifically that the high latitude Southern Ocean Site 1096 (see Fig. 1) displays similar behaviour as the North Pacific in terms of the long-term change with Northern hemisphere glaciation. This point also applies to the discussion on Lns 261-263, Lawrence et al., 2013 discuss this from a global perspective.

We thank the reviewer for bringing this to our attention and completely agree on integrating the valuable synthesis paper by Lawrence et al., 2013. We now include the link to productivity changes in the Antarctic Zone based on opal mass accumulation rates (ODP Site 1096) that strongly diminish at the intensification of the Northern Hemisphere Glaciation. This record has been interpreted as indicating the onset of high latitude stratification around Antarctica coeval to stronger stratification in the North Pacific (ODP Site 882) (Sigman et al., 2004; Nature). These results have been subsequently extended and it has been proposed that the decline in opal deposition is primarily indicating extended and prolongated sea-ice cover and, secondly, enhanced ocean stratification (Hillenbrand & Cortese, 2006; Paleo^3). We now added ODP Site 1096 opal MAR to Fig. 4 and discuss opal variations in the main text (lines 223-232).

Regarding the connection between 400kyr ACC changes and intermediate / deep-water formation in the subarctic North Pacific. Ln 252 states that "This phase of strongest PMOC as recorded in the opal deposition record from ODP Site 882" Note that while the opal record at 882 is interpreted by Burls et al. 2017 as a record for deep wintertime convection that reached down towards the depth of maximum silicate concentration (~1700 m in the modern North Pacific), the record for calcium carbonate accumulation rate is interpreted as a better measure of the strength of deep water formation, and by extension the PMOC, with high accumulation rates when the deep water formation ventilated all the way down to the depth of

the sea floor at the site. These records are similar (Fig 1c vs 1d in Burls et al., 2017) but this should be kept in mine for the interpretation discussed on Lns 254-259.

We thank Referee #1 for this suggestion. We replaced the opal record with carbonate accumulation rates (Figure 4) as a better record of North Pacific deep-water formation and, by extension, the PMOC (Burls et al., 2017; Sci. Adv.). Both the opal and carbonate records show elevated values culminating during the *Plio1* ACC phase indicating the decline of winter convection and deep-water formation. The Pliocene maxima in the $CaCO_3$ MAR record are partly different from the opal MAR record. For example, an early carbonate maximum corresponds to the early Pliocene ACC maximum *Plio3* and the late Pliocene occurs earlier than for the opal MAR.

Lns 282-289 – The mechanism behind the link between ACC strength and global delta13C stack needs to be fleshed out. Why and how they are linked is not clear in the current discussion.

We show the 400-kyr cycles in the global marine $\delta^{13}C$ stack in order to hint to changes in the global carbon reservoir which could be linked to Southern Ocean changes as evidenced in the ACC. However, there are several other factors affecting the marine carbon reservoir including changes in chemical weathering (e.g., de Boer et al., 2014). We think that a more thorough discussion of the global marine $\delta^{13}C$ stack changes would probably be beyond the scope of our paper. We keep the record in ED Fig. 1 to illustrate that the phasing of changes in the marine carbon reservoir in the ~400-kyr band likewise changes with the intensification of the Northern Hemisphere Glaciation and all other changes described in our general response above.

ACC, ocean fronts, and AIS development. From Lns 293-295, it is not clear what one should be looking at in Fig 4 to support this statement. Perhaps I missed it but what is your proxy for AIS developments in Fig. 4? Is there a record connecting AIS advances with reduced southward advection and upwelling of the CDW, north-ward shifted fronts/SSW, and your records of ACC. If not, this part of the discussion feels very speculative.

We apologize for the incorrect link to the previous Fig. 4 and fully agree with all three referees´ suggestion to graphically link our ACC strength record to Antarctic ice-sheet reconstructions. In the revised Fig. 4, we have now plotted the major advances and retreats of the West Antarctic Ice Sheet as derived from the ANDRILL sediment record (Naish et al., 2009; Nature). We compare this record also to the modelled ice changes by Pollard et al. (2009; Nature) and a modelled Antarctic ice-sheet record by deBoer et al. (2014; Nat. Comm.) (Fig. 4b). During the Pliocene, the overall reduced ice-sheet extent shows comparatively small advances that largely fall into intervals of reduced ACC. The strong WAIS ice volume increase deduced

from the ANDRILL record occurs with the iNHG and parallels the major weaking in our ACC record. We have added this to the discussion in lines 311-332.

References cited

Burls, N. J., & Fedorov, A. V. (2014). Simulating Pliocene warmth and a permanent El Niño-like state: The role of cloud albedo. Paleoceanography, 29(10), 893-910.

Boer, B. de, Lourens, L. J. & Wal, R. S. W. van de. Persistent 400,000-year variability of Antarctic ice volume and the carbon cycle is revealed throughout the Plio-Pleistocene. Nat Commun ***5****, 2999 (2014).*

Hillenbrand, C.-D. & Cortese, G. Polar stratification: A critical view from the Southern Ocean. Palaeogeogr., Palaeoclim., Palaeoecol. ***242****, 240–252 (2006).*

Hogg, A. M. (2010). An Antarctic Circumpolar Current driven by surface buoyancy forcing. Geophysical Research Letters, 37(23).

Johnson, G. C., & Bryden, H. L. (1989). On the size of the Antarctic Circumpolar Current. Deep Sea Research Part A. Oceanographic Research Papers, 36(1), 39-53.

Lawrence, K. T., Sigman, D. M., Herbert, T. D., Riihimaki, C. A., Bolton, C. T., Martinez-Garcia, A., ... & Haug, G. H. (2013). Time-transgressive North Atlantic productivity changes upon Northern Hemisphere glaciation. Paleoceanography, 28(4), 740-751.

Naish, T. et al. Obliquity-paced Pliocene West Antarctic ice sheet oscillations. Nature ***458****, 322–328 (2009).*

Nie, J. et al. Pacific freshening drives Pliocene cooling and Asian monsoon intensification. Sci. Rep. ***4****, 5474 (2014).* https://dx.doi.org/10.1038/srep05474

Pollard, D. & DeConto, R. M. Modelling West Antarctic ice sheet growth and collapse through the past five million years. Nature ***458****, 329–332 (2009).*

Sigman, D. M., Hain, M. P. & Haug, G. H. The polar ocean and glacial cycles in atmospheric CO2 concentration. Nature ***466****, 47–55 (2010).*

Toggweiler, J. R., Russell, J. L., & Carson, S. R. (2006). Midlatitude westerlies, atmospheric CO2, and climate change during the ice ages. Paleoceanography, 21(2).

Minor comments:

Title. What "emergence of coupled" meant in the title only made sense to me after reading the manuscript. I wonder if this can be reworded?

Following the reviewers' and the editor's suggestion we changed the title to "Five million years of Antarctic Circumpolar Current strength variability"

Abstract. Define "Long-term"

We agree that the term "long-term" needs a more specific definition. We now define "long-term" as: (1) the linear trend over the whole length of our records (i.e., 5.3 Ma). (2) we now also discuss a one-million-year smoothed ACC strength record showing the contrast of global climate development (Lisiecki and Raymo benthic $\delta^{18}O$ record) and our ACC strength record.

Abstract. "Antarctic ice volume simulations 11 and precessional changes in the South Pacific Jet linked to tropical Pacific temperature changes 12" -> the link to tropical Pacific temperature changes is not explicitly discussed in the discussion on Lns 239-248 so it is surprising to see this statement in the abstract.

We corrected this and revised the discussion on the split jet and the link to zonal SST changes in the tropical Pacific (lines 258-260).

Ln 95 “small variations in ACC strength documented” -> are documented

Thank you, corrected.

Lns 95-97 I found this sentence hard to follow

Thank you for pointing out that this rather long sentence is hard to follow. We have revised the text accordingly

Ln 181 “Compared our” -> “Compared to our”

Thank you, corrected.

Ln 266-267 What do the authors mean by “diffuse deep PMOC”

We agree and deleted “diffuse”.

Ln 370 e. -> f.

Thank you, corrected.

Response to Referee #2 (Oceanography; Sortable Silt):

(comments by the reviewer are printed in italics, our response in blue)

A. This is a most interesting and potentially significant paper dealing with variations in the ACC flow strength in the central South Pacific.

We thank the reviewer for their very positive assessment of our manuscript.

However it is not clear that the word 'Emergence of' in the title is warranted because significant variations in ACC strength have occurred since at least 5 Ma with a strong pulse at that point in time. And while there is a clear trend in global climate cooling since the Pliocene there is no such trend in the ACC flow strength, a point made in the opening paragraph. The important point is that ACC strength does NOT match global cooling since the Pliocene.

We fully agree with this statement. We have now substantially expanded the discussion on the "long-term" trends across the entire 5.3 Myr time-series (linear trend) by discussing a one-million-year smoothed ACC strength record and linking the observed trends and changes to key paleoclimatic changes across the Plio-Pleistocene.

We thank Referee #2 for their critical evaluation of the long-term Plio-/Pleistocene trend in our records and potential forcing mechanisms. These very relevant comments are complementary to other referees' comments and we address these points in more detail in the main text and in this letter.

In the original manuscript, we referred to the absence of a linear "long-term trend" across the entire time-series (i.e., 5.3 Ma), in contrast to the linear trends in global climate (e.g., the Lisiecki/Raymo $\delta^{18}O$ stack and Pacific SST records) and ice volume over this time period. However, the overall Plio-Pleistocene climate evolution from warmer-than-present early Pliocene conditions (with reduced meridional SST gradients and small Antarctic ice-sheets/sea-ice extent) to the Pleistocene bi-polar glaciation with enhanced ocean-atmospheric gradients ostensibly implies strengthening of the westerly winds together with Southern Ocean density gradients, both of which would enhance the ACC. This overall configuration is challenging to reconcile with the missing long-term linear trend in our ACC strength time-series.

Inspired by the referees, we therefore re-assessed the "long-term" trends in light of other feasible forcing mechanisms for ACC strength changes over the past 5.3 Ma. Whereas there is no linear long-term trend over the entire length of the record (as stated in our original manuscript), there are trends beyond the ~400 kyr cycles. The one million-year-smoothed ACC strength record reveals a long-term increasing trend throughout the Pliocene cooling trend (Fig. 4). In contrast to the Pliocene, during the early Pleistocene (until ~1.5 Ma), decreasing ACC strength parallels global climate cooling and ice volume increase. Thereafter ACC flow increases again towards the Mid-Pleistocene-Transition (MPT).

In order to explain these contrasting trends, we propose that the ACC responded to fundamentally different forcings during the Pliocene compared to the Pleistocene. The shift in the ACC regime occurred with the major climate reorganisation connected to the intensification of the Northern Hemisphere Glaciation (iNHG) that was accompanied by major atmosphere-ocean circulation changes and increasing Antarctic ice-sheets and sea-ice extent.

During the Pliocene, the absence of a major marine-based Antarctic ice-sheet, the strongly reduced sea-ice cover, and weakened density gradients across the Southern Ocean compared to the Pleistocene, led to less developed oceanic fronts. Such a Pliocene Southern Ocean setting would have strengthened the sensitivity of the ACC to atmospheric forcings compared to oceanic forcings. The Pliocene increasing trend in ACC strength can be plausibly explained by overall increasing atmospheric forcing, consistent with a progressive equatorward movement and intensification of the westerlies in response to decreasing global temperatures, increasing latitudinal temperature gradients, and growing development of Southern Ocean density gradients. The Pliocene atmosphere-ocean changes also parallel the initiation of zonal gradients across the Eastern Pacific cold tongue and increasing Asian monsoon from ~4.2 to 2.75 Ma (Fig. 4). Together, these changes plausibly contributed to enhanced wind forcing of the Pacific ACC through teleconnections involving the southern Hadley cells and the southern westerlies. It is conceivable that Pliocene tectonic changes, such as uplift of the Tibetan Plateau affected gradual trends in the Asian monsoon (Nie et al., 2014; Sci Rep.). However, models do not suggest a major impact of the final closure of the Central American Seaway on large-scale Pacific circulation, including ACC strength (e.g. Burls et al., 2017, Sci. Adv.).

The shift from the Pliocene to the Pleistocene ACC response occurred after the late Pliocene ACC maximum *Plio 1* with a major decrease of ACC strength. This change is supposedly connected to the major global climate shift involving the iNHG, and a further step in the development of Antarctic ice-sheets, particularly the West Antarctic Ice Sheet (WAIS) (McKay et al., 2012; PNAS). We suggest that major changes around Antarctica, e.g., sea-ice extent and deep-water formation, strengthened the latitudinal ocean density gradients, thereby enhancing Southern Ocean fronts and displacing them northward. This scenario in all likelihood explains the concomitant increase in opal deposition at our sites and the decrease in the Antarctic Zone (e.g., at ODP Site 1096) (now plotted in Fig. 4k).

In the revised manuscript (lines 209 to 221), we now discuss that this fundamental reconfiguration of the Southern Ocean altered the sensitivity of the ACC to the different atmospheric and oceanic forcings. In contrast to the Pliocene, during the early Pleistocene (until ~1.5 Ma), global climate cooling and ice volume increase is paralleled by a multi-million-year trend of decreasing ACC strength. This is in contrast to the expected increasing ACC strength due to stronger atmospheric and oceanic gradients. As pointed out by the referees concerning the long-term trend along the entire record, the early Pleistocene pattern of opposite changes in global temperature/ice volume and ACC strength requires compensating mechanisms resulting in a net decrease of ACC strength.

We propose two major factors:

(a) Changes in ocean density gradients: We propose that after global cooling along with the intensification of the NHG and the build-up of Antarctic ice-sheets, ocean temperatures in the Antarctic Zone approached the freezing point and thus further Southern Ocean cooling decreased meridional temperature gradients in the southern mid-latitudes (and did not not enhance these gradients as would have occurred during the Pliocene). Thus, latitudinal density gradients became weaker across the Southern Ocean fronts with progressive cooling. The same mechanism ostensibly contributes to glacial-interglacial changes with the weaker ACC during glacials (e.g., Lamy et al., 2019; PNAS).

(b) Changes in atmospheric forcings related to the South Pacific Split Jet. In our original manuscript, we discuss the ~400-kyr cycles in terms of long-term changes in the configuration the Pacific branches of high-altitude atmospheric jets affecting the wind forcing of the ACC. In this concept, a reduced split jet enhances ACC wind stress forcing as shown by modelling studies (e.g., Chiang et al., 2018; Lamy et al., 2019). Changes of the split jet are linked to zonal SST gradients in the tropical Pacific and the strength of the Asian monsoon. Decreased zonal gradients and stronger monsoon weaken the split jet and strengthen the subantarctic westerlies in the Pacific sector, and thus the ACC. This mechanism is also consistent with the long-term Pliocene enhancement of the monsoon and the ACC.

After the long-term ACC strength minimum at ~1.5 Ma, the one million-year-smoothed ACC strength record increases again, coinciding with a major SST cooling in the subarctic and subantarctic zones (Martinez-Garcia et al., 2010). This cooling and connected strengthening of atmospheric circulation substantially increased dust supply to the South Atlantic towards the MPT (Martinez-Garcia et al., 2011). These patterns are consistent with the strong glacial-interglacial amplitudes of the ACC strength with the beginning of the MPT at ~1.3 and again at ~1 Ma.

We have added a respective part to the main text describing the trends and proposed mechanistic links in section “Long-term ACC development”, lines 209 to 260.

B. Methods are not novel but the location is of the highest importance and the length of the record is far greater than any other.

Thank you, we appreciate this statement.

C. This paper employs measurements of the sortable silt flow strength proxy and transfers them to the elemental ratio ln(Zr/Rb) for which there is are detailed records from XRF core scanning for the last 5.3 million years. The correlation on which this is based (extended data figure 9b) shows considerable scatter with r2 of only 0.42. A point that the authors will need to answer is that several of them, including Wu and Lamy, had a paper at the 2022 Bergen Paleoceanography conference dealing with the ACC in Drake passage which casts some doubt on the universality of the Zr/Rb relation to flow strength (Wu et al., 2022).

Because in an earlier paper they had found coherent changes in sortable silt and Zr/Rb ratios, but in the work reported in the Bergen abstract the opposite was found, they said “variations of Zr/Rb ratio may thus have been primarily controlled by changes in weathering intensity”. Clearly there are several controls of sediment compositional variations, of which flow speed control of grain size is one but climate-controlled weathering is another. Nevertheless, the relationship between sortable silt mean size and Zr/Rb ratio shown in extended data figure 9a is quite convincing.

We agree with Referee #2 that the correlation of ln(Zr/Rb) and sortable silt is relatively low, compared to previous studies. However, most of the prior studies were located at or relatively proximal to continental margins with sediments consisting mainly of siliciclastic components. This renders grain-size measurements less susceptible to incomplete removal of carbonates and biogenic opal (opal contents are generally low in most previous studies), and strongly differs from our setting with biogenic (carbonate and opal) sedimentation predominating. Therefore, we suspect that part of the scatter and the resulting relatively low correlation coefficient might be related to incomplete removal of biogenic components (though we controlled for removal of carbonate and opal with the microscope after chemical treatment). Further scatter might be introduced by the laser-particle sizes method applied, because the remaining siliciclastic fraction was too small to be analysed on a Sedigraph, which would be otherwise the preferable instrument for such analyses as it considers the dynamical behaviour of the sediment grains (McCave et al., 2006).

We are aware that other factors, such as continental weathering, might affect the Zr/Rb composition as a proxy for sortable silt and bottom current speed. The referee correctly mentioned the ongoing work by Shuzhuang Wu and colleagues in the Indian Ocean, that was presented at ICP in Bergen and also at the Goldschmidt Conference in Lyon, showing a substantial impact of continental weathering on Zr/Rb. However, given the difference in location, we conclude that, if a weathering influence would at all affect our central South Pacific records, this effect would be minor, given the large distance to any continent with substantial chemical weathering (in contrast to the Indian Ocean). Additional support comes from the records from the Southeast Pacific off Chile and the Drake Passage which provide excellent correlations of Zr/Rb to the mean sortable silt. We address this point in the Methods section.

We thank Referee #2 for acknowledging that the relationship between sortable silt and Zr/Rb shown in ED Figure 9b is quite convincing.

The authors refer to assessment of flow speeds following calibrations by McCave et al. (2017) but do not give the equation used (line 133 and extended data line 598). The point at issue here is that in February many users of the sortable silt proxy received a notification from McCave that the equations in a table in that paper were incorrect but those on the figure were good. It would be helpful to know whether Lamy et al. used the correct equation here.

The sortable silt measurements are plotted against age in extended data figure 9a but it is not stated from which core the samples were taken.

We thank Referee #2 for referring to the issue with the correction of McCave's 2017 work. We used the correct equation from Fig. 3 in McCave's work from the Scotia Sea (McCave et al., 2017; QSR) which is now explicitly mentioned in the Methods section

D. Uncertainies in transfer of ln(Zr/Rb) to grainsize and hence flow speed. not treated.

The uncertainties in transferring ln(Zr/Rb) to sortable silt are related to the grain-size measurements, as the analytical error of the XRF Core Scanner for both Zr and Rb is small. We have not measured these elements wet-chemically but the signal to noise ratio for both elements has been previously shown to be excellent as those "heavy elements are precisely detected by X-ray fluorescence, even at low concentrations (Wu et al., G-cubed, 2020). As discussed above, the grain-size measurements are comparatively noisy at our site

(U1541) but the overall slope of the linear equation used for the calculation of sortable silt is sufficiently defined.

To our knowledge, there are no modern grain-size data and instrumental current meter data available from our working area. Therefore, we use the Scotia Sea calibration of McCave et al. (2017), one of few available from the Southern Ocean. Alternatively, we could have used the Wu et al. (2019) equation based on data from the Drake Passage but that equation might be less suitable for the open ocean ACC. We therefore do not discuss in detail the absolute flow speeds but rather the relative deviation from the Holocene mean in the main text (see Methods section and additional Extended Data Figures 8-10).

Taken together, we emphasize that – given the underlying linear relationships - the relative variations of the reconstructed ACC flow speed in the central South Pacific (i.e., the consistent shape and amplitude of ACC variations at different scales and sites) are not affected by the transfer steps from Zr/Rb to current strength.

E/F. Lines 250 271, paragraph #14, contains the unresolved contrast between ACC and PMOC strength during Plio- 1 and the anti-phase Plio2 and 3 and continues with rather weak speculation concerning relationships between the North and South Pacific.

Yes, this contrast has been correctly observed by Referee #2. Indeed, as suggested also by Referee #1, we now focus on the carbonate accumulation-rate record from ODP Site 882 as a more suitable PMOC record. This record is more in phase during ACC maximum *Plio3* but suggests a strong PMOC over a more extended late Pliocene intervals with an earlier decrease than the opal record (see discussion in Burls et al., 2017; Sci. Adv.). We now include the carbonate accumulation rate record from ODP Site 882 in Fig. 4

The next paragraph, (#15, 273-289), is also in the nature of a general discussion based mainly on previously published information. There are matters of importance in here; for example why does the ACC strength display a changing phase in relation to the carbon isotope stack, a point which is mentioned but not explained (see extended figure 8).

We now extended the discussion of the ~400-kyr cycles by comparing (in our new ED Fig. 5) to the filtered records of Antarctic ice-volume (modelled by de Boer et al., 2014; NComm), the Asian Monsoon record (Sun et al., 2010; EPSL) and a global marine $\delta^{13}C$ stack (as previously shown in ED Fig. 8). The ~400-kyr-

filtered records allow for extended insight into changes at this spectral band which is strongly observed in the ACC strength record. For example, band-pass filtered ACC record and Antarctic ice volume records are mostly anti-phased over the Pliocene and early Pleistocene, consistent with the expected link between a stronger ACC and ice-sheet retreat (see discussion in our manuscript, lines 311-332, ED Fig. 8c and d). Likewise, there is a relationship between stronger monsoon and reduced ACC strength (ED Fig. 11c and e) which is contrary to the Pliocene multi-million-year trend and Pleistocene glacial/interglacial variations.

The next paragraph (#16, 291-310) relates important connections between fluctuations of the West Antarctic ice sheet (increases) to variations in ACC strength (decreases). However figure 4 referred to on line 299 does not contain any information regarding fluctuations in the WAIS.

We apologize for the incorrect link to the previous Fig. 4 and fully agree with all three referees´ suggestion to graphically link our ACC strength record to Antarctic ice-sheet reconstructions. In the revised Fig. 4, we now plotted the major advances and retreats of the WAIS as derived from the AND-1B sediment record (Naish et al., 2009; Nature). We compare this record also to the modelled ice-changes by Pollard et al. (2009; Nature) and a modelled Antarctic ice-sheet record by deBoer et al. (2014; NComm.) (Fig. 4b). During the Pliocene, the overall reduced ice-sheet extent shows comparatively small advances that overall fall into intervals of reduced ACC. The strong WAIS ice volume increase deduced from the AND-1B record occurs with the intensification of the NHG and parallels the major weaking in our ACC record. We have added this to the discussion in lines 311-332.

The remainder of the paragraph also contains several rather weak statements relating climate cycles, ice sheet cyclicity.

Paragraph #17 is a coda that draws attention to the likely consequence of a warming climate being an increase in ACC flow strength.

Overall the discussion paragraphs 14 to 17 (250-324) are rather more speculative and less tightly tied to the database presented here than would be expected in a paper for Nature, and more commonly associated with a journal such as Paleoceanography.

We agree with the reviewer that the previous paragraphs required strengthening in presentation and underlying mechanisms. To address this, we have tightened up our mechanistic discussion by clarifying the roles of atmospheric forcing (through the South Pacific Split Jet) and oceanographic forcing (through density contrasts across the Southern Ocean fronts) throughout the manuscript (see first response section). As discussed above, we have additionally modified Figure 4 and ED Fig. 8 to more directly link our new ACC strength reconstruction with relevant paleoclimate records speaking to Antarctic Ice Sheet extent,

Southern Ocean frontal migration, and PMOC. We believe the final paragraph 17 is warranted as our data greatly expands the paleoclimate support for strengthening ACC flow with a continued warming climate.

Line comments.

133. Check that the correct equation following McCave's correction notice has been used .

See our response to a similar comment above. We used the correct equation and specified this in the figure caption of ED Fig. 7.

135-6. It would be helpful to clarify here that the density is a proxy for opal (because diatoms are hollow) and the calcium counts are a proxy for carbonate.

We thank the Referee for this comment and added the information to supplementary material

153. The ACC strength is frequently referred to as a percentage in figure captions; it would be helpful if, on first use in figure 2d, the percentage referred to is specified as the ratio of ln(Zr/Rb) to its Holocene mean (if my understanding is correct). Is Holocene here 0-11.7 ka?

We added this information to the Methods section

164. Specify figure 2e. In several figure references later, particularly regarding figure 4, the letter designations of the panels are not given.

We refer to Fig. 4 in cases more than one panel is meant, otherwise we add the letter to point to a specific record.

166. Why is the ACC here referred to as pelagic? Is this to distinguish it between the constrained ACC in the Drake passage, if so the point is obscure and the word could well be omitted.

We agree and deleted “pelagic”.

181. ... Compared to our ...

211. After 3.8 Ma refer to figure 4f.

217 4c

223 4d

241. ....invoked not involved

263, 4d

346. Insert the meaning of percentage in ACC strength as suggested above.

370. e on this line should be f.

546. The stratigrapher was improved by ‘correlating’ not by ‘tuning’.

We thank the reviewer for the careful revisions and corrected the respective text parts accordingly.

590-2. The point about the weathering control of the Zr/Rb ratio in Wu et al 2022 needs to be addressed here. It might also be worthwhile to include the definition of percentage strength as a percentage of the Holocene mean and specify what that value is and over what period it is averaged.

We now define the Holocene mean more clearly in the methods section

626. The red line in the upper part of the figure needs to be labelled STF and the caption needs to have subtropical front (STF) inserted. Because the fronts move about 5° further north during glacials the authors might say something about the relationship between U1541 and U1540 and the likely glacial positions of the fronts.

We corrected the missing caption “STF”. We agree that most likely closer or potentially south of the SAF. However, glacial/interglacial frontal shifts could be limited by fracture zones (Park et al., 2019).

629 and 643. The fonts in these figure axis labels are too small.

663. Insert a 'b' for the second panel.

675. Insert a scale and label for the right-hand axis of panel a.

694, 697. Incorrect capitalisation.

We thank the reviewer for the careful revisions and corrected the manuscript.

G. see refs to references above.

We do not understand

H. From lines 250 to 324 could be tightened up and less speculative.

See response to comment on this above.

References used

McCave, I. N., Thornalley, D. J. R. & Hall, I. R. Relation of sortable silt grain-size to deep-sea current speeds: Calibration of the 'Mud Current Meter.' Deep Sea Research Part I: Oceanographic Research Papers ***127****, 1–12 (2017).*

Park, Y. -H. et al. Observations of the Antarctic Circumpolar Current Over the Udintsev Fracture Zone, the Narrowest Choke Point in the Southern Ocean. J. Geophys. Res.: Oceans ***124****, 4511–4528 (2019).*

Sun, Y., An, Z., Clemens, S. C., Bloemendal, J. & Vandenberghe, J. Seven million years of wind and precipitation variability on the Chinese Loess Plateau. Earth Planet Sc Lett ***297****, 525–535 (2010).*

Wu, L. et al. Evaluating Zr/Rb Ratio From XRF Scanning as an Indicator of Grain-Size Variations of Glaciomarine Sediments in the Southern Ocean. Geochem Geophys Geosystems ***21****, (2020).*

Response to Referee #3 (ACC):

(comments by the reviewer are printed in italics, our response in blue)

Review of the manuscript "Emergence of coupled Antarctic Circumpolar Current flowstrength and global climate cooling over the last 5 million years" by Lamy and co-authors

This manuscript uses (i) high-resolution XRF-based Zr/Rb ratios, calibrated with discrete sortable silt data; (ii) high-resolution biogenic opal contents, obtained from polynomialregression between GRA-density and discrete biogenic opal measurements; (iii) high-resolutionCaCO3 contents obtained from XRF-based Sr count data, calibrated with the discrete CaCO3content measurements and (iv) benthic foraminifera oxygen and carbon isotope data from two pelagic sediment cores in the Central South Pacific Ocean, IODP sites U1540 and U1541.Together with sites U1540 and U1541, three piston cores (PS75/76, PS75/79, PS75/83) were also studied in order to monitor ACC changes along a north-south transect in the Pacific sector of the Southern Ocean since the ~5.3 Ma.

The authors document a close link of ACC strength to the orbital eccentricity cycle with a period of 400,000 years and propose that this is likely originated from the modulation of precessional changes in the South Pacific jet stream linked to tropical Pacific temperature changes. Moreover, they suggest a stronger Pacific-wide circulation during the warm late Pliocene. They also report a link between weaker ACC flow, an equatorial extended opal belt and reduced CO2 in glacial intervals first emerged during the Mid-Pleistocene Transition and persisted for the last 800 kyr. Lastly, they show that the strongest ACC flow occurred during warmer-than present intervals of the Pliocene and Pleistocene.

Overall, the paper provides new important dataset for the past evolution of the ACC. Given the importance of the ACC for the global climate, these results are original and of major importance. However, there are concerns on some of the interpretations and mechanisms proposed that Ifeel need to be addressed before publication. I have elaborated further below where I had key questions over the interpretation, but in summary I believe that more evidence is required to support some of the proposed mechanisms and conclusions.

We thank Referee #3 for their positive evaluation of our manuscript.

I believe that the current title of the manuscript is not capturing the main message of the paper.I would suggest that the authors consider changing it.

We agree with the referee and changed the title to "*Five million years of Antarctic Circumpolar Current strength variability*".

Lines 92-100: Given that several records along the Southern Ocean point to regional heterogeneity of ACC flow in the different sectors of the Southern Ocean over Pleistocene glacial-interglacial cycles, and your records do not show a long-term trend in ACC flow, following the gradual global cooling and increase in global ice-volume, I am not convinced(however do not completely disagree) that a north-south transect across ACC in the Pacific sector of the Southern Ocean could really provide insights on the overall ACC fluctuations. Is it possible that you record a more regional view of the dynamics of the ACC?

The consistent glacial-interglacial fluctuations in the shorter cores forming the cross-frontal meridional transect across the entire ACC (and across a broad range of oceanic and sedimentary conditions) suggests a representative ACC strength pattern in the open ocean Pacific. Moreover, the central South Pacific records are also consistent with other Pacific records from the Drake Passage region, all showing ACC strength decrease during glacials compared to interglacials. Considering that the Pacific Southern Ocean is by far the largest sector of the ACC, we think that our records are representing a large fraction of overall ACC variability. We discuss and acknowledge potential heterogeneities in other ACC sectors for the last glacial-interglacial cycles on Lines 90-98, but we emphasize that there are no equivalent ACC records in the Indian and Atlantic sectors extending to the Pliocene. We also present in the revised manuscript a conceptual model how the seemingly contradictory between ACC strength and large-scale climate forcings can be reconciled (see next point).

As you mention in lines 105-108: reconstructions of southern hemisphere meridional SST gradients indicate an overall strengthening of the atmospheric circulation (SWW) over the Pliocene and early Pleistocene, that would most plausibly imply an enhancement of the ACC. So, why you do not see an overall long-term increase in ACC flow speeds in the Central Pacific? I think this is an interesting finding that needs further explanation.

We agree and provide an extensive discussion of this point. We acknowledge that this has been a major point in the reviews by all referees. We now provide a mechanistic link and discuss the variability of our ACC strength record in the context of various forcings.

In the original manuscript, we referred to the absence of a linear "long-term trend" across the entire time-series (i.e., 5.3 Ma), in contrast to the linear trends in global climate (e.g., the Lisiecki/Raymo $\delta^{18}O$ stack

and Pacific SST records) and ice volume over this time period. However, the overall Plio-Pleistocene climate evolution from warmer-than-present early Pliocene conditions (with reduced meridional SST gradients and small Antarctic ice-sheets/sea-ice extent) to the Pleistocene bi-polar glaciation with enhanced ocean-atmospheric gradients ostensibly implies strengthening of the westerly winds together with Southern Ocean density gradients, both of which would enhance the ACC. This overall configuration is challenging to reconcile with the missing long-term linear trend in our ACC strength time-series.

Inspired by the referees, we therefore re-assessed the "long-term" trends in light of other feasible forcing mechanisms for ACC strength changes over the past 5.3 Ma. Whereas there is no linear long-term trend over the entire length of the record (as stated in our original manuscript), there are trends beyond the ~400 kyr cycles. The one million-year-smoothed ACC strength record reveals a long-term increasing trend throughout the Pliocene cooling trend (Fig. 4). In contrast to the Pliocene, during the early Pleistocene (until ~1.5 Ma), decreasing ACC strength parallels global climate cooling and ice volume increase. Thereafter ACC flow increases again towards the Mid-Pleistocene-Transition (MPT).

In order to explain these contrasting trends, we propose that the ACC responded to fundamentally different forcings during the Pliocene compared to the Pleistocene. The shift in the ACC regime occurred with the major climate reorganisation connected to the intensification of the Northern Hemisphere Glaciation (iNHG) that was accompanied by major atmosphere-ocean circulation changes and increasing Antarctic ice-sheets and sea-ice extent.

During the Pliocene, the absence of a major marine-based Antarctic ice-sheet, the strongly reduced sea-ice cover, and weakened density gradients across the Southern Ocean compared to the Pleistocene, led to less developed oceanic fronts. Such a Pliocene Southern Ocean setting would have strengthened the sensitivity of the ACC to atmospheric forcings compared to oceanic forcings. The Pliocene increasing trend in ACC strength can be plausibly explained by overall increasing atmospheric forcing, consistent with a progressive equatorward movement and intensification of the westerlies in response to decreasing global temperatures, increasing latitudinal temperature gradients, and growing development of Southern Ocean density gradients. The Pliocene atmosphere-ocean changes also parallel the initiation of zonal gradients across the Eastern Pacific cold tongue and increasing Asian monsoon from ~4.2 to 2.75 Ma (Fig. 4). Together, these changes plausibly contributed to enhanced wind forcing of the Pacific ACC through teleconnections involving the southern Hadley cells and the southern westerlies. It is conceivable that Pliocene tectonic changes, such as uplift of the Tibetan Plateau affected gradual trends in the Asian monsoon (Nie et al., 2014; Sci Rep.). However, models do not suggest a major impact of the final closure of the Central American Seaway on large-scale Pacific circulation, including ACC strength (e.g. Burls et al., 2017, Sci. Adv.).

The shift from the Pliocene to the Pleistocene ACC response occurred after the late Pliocene ACC maximum *Plio 1* with a major decrease of ACC strength. This change is supposedly connected to the major global climate shift involving the iNHG, and a further step in the development of Antarctic ice-sheets, particularly the West Antarctic Ice Sheet (WAIS) (McKay et al., 2012; PNAS). We suggest that major changes around Antarctica, e.g., sea-ice extent and deep-water formation, strengthened the latitudinal ocean density gradients, thereby enhancing Southern Ocean fronts and displacing them northward. This scenario in all likelihood explains the concomitant increase in opal deposition at our sites and the decrease in the Antarctic Zone (e.g., at ODP Site 1096) (now plotted in Fig. 4k).

In the revised manuscript (lines 209 to 221), we now discuss that this fundamental reconfiguration of the Southern Ocean altered the sensitivity of the ACC to the different atmospheric and oceanic forcings. In contrast to the Pliocene, during the early Pleistocene (until ~1.5 Ma), global climate cooling and ice volume increase is paralleled by a multi-million-year trend of decreasing ACC strength. This is in contrast to the expected increasing ACC strength due to stronger atmospheric and oceanic gradients. As pointed out by the referees concerning the long-term trend along the entire record, the early Pleistocene pattern of opposite changes in global temperature/ice volume and ACC strength requires compensating mechanisms resulting in a net decrease of ACC strength.

We propose two major factors:

(a) Changes in ocean density gradients: We propose that after global cooling along with the intensification of the NHG and the build-up of Antarctic ice-sheets, ocean temperatures in the Antarctic Zone approached the freezing point and thus further Southern Ocean cooling decreased meridional temperature gradients in the southern mid-latitudes (and did not not enhance these gradients as would have occurred during the Pliocene). Thus, latitudinal density gradients became weaker across the Southern Ocean fronts with progressive cooling. The same mechanism ostensibly contributes to glacial-interglacial changes with the weaker ACC during glacials (e.g., Lamy et al., 2019; PNAS).

(b) Changes in atmospheric forcings related to the South Pacific Split Jet. In our original manuscript, we discuss the ~400-kyr cycles in terms of long-term changes in the configuration the Pacific branches of high-altitude atmospheric jets affecting the wind forcing of the ACC. In this concept, a reduced split jet enhances ACC wind stress forcing as shown by modelling studies (e.g., Chiang et al., 2018; Lamy et al., 2019). Changes of the split jet are linked to zonal SST gradients in the tropical Pacific and the strength of the Asian monsoon. Decreased zonal gradients and stronger monsoon weaken the split jet and strengthen the

subantarctic westerlies in the Pacific sector, and thus the ACC. This mechanism is also consistent with the long-term Pliocene enhancement of the monsoon and the ACC.

After the long-term ACC strength minimum at ~1.5 Ma, the one million-year-smoothed ACC strength record increases again, coinciding with a major SST cooling in the subarctic and subantarctic zones (Martinez-Garcia et al., 2010). This cooling and connected strengthening of atmospheric circulation substantially increased dust supply to the South Atlantic towards the MPT (Martinez-Garcia et al., 2011). These patterns are consistent with the strong glacial-interglacial amplitudes of the ACC strength with the beginning of the MPT at ~1.3 and again at ~1 Ma.

We have added a respective part to the main text describing the trends and proposed mechanistic links in section "Long-term ACC development", lines 209 to 260.

Line 108: You could consider adding a more recent reference - SST record offshore Tasmania(IODP Site 1168) (Hou et al. 2023; https://doi.org/10.5194/cp-19-787-2023)

We thank the reviewer for pointing to this more recent study on SST changes in vicinity of the Subtropical Front of Tasmania. The overall Plio-Pleistocene cooling trend in these data is consistent with the Federov et al. (2015) data we cited. However, Hou et al., focus more on the Miocene at a site that might locally amplify Pliocene SST variations through the influence of the changing Leeuwin Current and SST changes at the STF itself.

Line 110: I would delete the word "position". Although you document shifts of the ACC frontal system, you do not reconstruct the position of the ACC during the study interval.

This is correct for the two Plio/Pleistocene ACC records from IODP Sites U1540 and U1541. However, our cross-frontal transect addresses also the question of changes in the position over the past few glacial/interglacial cycles.

We therefore delete the word "position" as the sentence refers to the entire period (5.3 Ma).

Lines 123: It would be very useful to show the depths of the piston cores too, as well as provide references associated to this information.

We added this information to the main text (lines 122-123) and at the beginning of the Methods section.

Line 153. You should mention which cores and the mean SS values you used to calculate theme an Holocene flow value (maybe add it in methods).

We added this information to the methods section.

Lines 170-171: "The spatial coherence of glacial decrease does not necessarily require large latitudinal 171 shifts of the ACC and SWW". Again, in lines 198-200, you suggest that the changes you observe in your opal % records do not necessarily require a substantial latitudinal shift of the opal belt. As you do not provide any data, this is rather speculative.

How can you differentiate from the low/high opal % shown in your data if they are controlled by lower/higher biogenic productivity or/and latitudinal migration of the frontal system and thus shifts of the opal belt?

Although not shown in the manuscript or in the supplementary data, I understand that you have opal and XRF Ca data from all sites (IODP and PS cores). Since you have a north south transect across the ACC frontal system, you could use them, together with diatom/calcareous counts (if available) to check if there were any substantial latitudinal migration. Good/bad preservation of microfossils can also affect your results. SEM images on siliceous and calcareous microfossils will help to strengthen your arguments. Saying that, I am a little confused when in lines 220-225: you suggest that the opposite variations between ACC strength and opal/carbonate ratios implies a northward extension of opal belt during glacials with reduced ACC strength. I think you need to provide stronger evidence on your interpretation on the opal % and opal/carbonate ratios.

We agree with Referee #3. The opal records shown in Fig 3 were focussing on the longer time-span including the Mid-Pleistocene Transition. We now added the opal data from the cross-frontal transect to the supplements (Extended Data Figs. 8-10). All cores show higher opal deposition during glacials. Only

the southernmost core PS75/83 is different. Here, in the Antarctic Zone, high opal values occur throughout the record but are moderately elevated during interglacials.

As mentioned in the text, we do not exclude latitudinal migrations of the fronts. We now modified the text (line numbers 197-202) in the direction that we conclude that the data can be interpreted in terms of overall movements of the fronts, changes in upwelling of nutrients and ocean stratification in the sea ice-free ACC, together with stronger glacial iron fertilisation through enhanced dust supply.

We thank Referee #3 for their suggestion that micropaleontological studies can provide additional information on frontal movements. Regarding diatoms, extensive work on sea-ice reconstructions for the last glacial maximum time slice has been previously published, including cores at or close to our locations (Benz et al., 2016) but counts have not been compared to counts of calcareous microfossil assemblages. $CaCO_3$ contents and XRF counts are available from survey cores as part of the proposal for the IODP Expedition 383. As an example, we here refer to the subantarctic site U1541 (data from survey core PS75/59; see Fig. 1) with moderate to high CaCO3 contents and comparatively low opal contents. Further south, carbonate contents decrease substantially with significant $CaCO_3$ contents occurring only during interglacials (Extended Data Fig. 8-10).

Lines 181-185: Why there is such as difference in the mean SS values between your sites and PS97/93 given that they are all at a similar depth?

Yes, mean sortable silt values are higher at site PS97/93 compared to U1541. We propose two main reasons for this. First, PS97/93 is located directly at the modern Subantarctic Front at the entrance of the Drake Passage. Sortable silt values are even higher within the Drake Passage as shown by Wu et al. (2021). Therefore, it is conceivable that bottom currents are indeed higher at site PS97/93 compared to U1541 north of the front. Second, there might be uncertainties regarding the correlation of ln(Zr/Rb) to sortable silt related to the different slope of the correlation in the Southeast Pacific compared to the Drake Passage (see discussion below).

Lines 205-214. As I mentioned before, this is a very interesting observation that I believe needsmore explanation. Why you do not see any long-term strengthening of the ACC following the global cooling and intensification of SWW? Does this suggest a local response rather than a more general ACC response?

We would like to point the Referee to our detailed response on the “long-term” ACC changes and varying forcing mechanism further up in our response letter.

Lines 259-271: I think that this section needs to be developed a bit more and specific connections are to be made between your data and available records. In this current version itis very speculative. Lines 294-300: This part was hard to follow as you cite figure 4 for the comparison between he WAIS retreats /advances and the variations in the ACC strength, but you do not show any data for the WAIS dynamics. Since you propose an important link between the ACC, frontal system and Antarctic ice sheet, I believe you should add available published data reporting WAIS dynamics in Fig.4.

We agree that this part would benefit from further explanation. We apologize to the Referee for including the incorrect link to Figure 4 in the original submission. We now graphically link our ACC strength record to Antarctic ice-sheet variability. In the new Fig. 4, we now plotted the major advances and retreats of the WAIS as derived as derived from the AND-1B sediment record (Naish et al., 2009; Nature). We compare this record also to the modelled ice-changes by Pollard et al. (2009; Nature) and a modelled Antarctic ice-sheet record by de Boer et al. (2014; Ncomm.) (Fig. 4b). During the Pliocene, the overall reduced ice-sheet extent shows comparatively small advances that overall fall into intervals of reduced ACC. The strong increase in the AND-1B record occurs with the intensification of NHG and parallels the major weaking in our ACC record.

Main Figures: There is a mistake in the colors between the legends and the data lines of the piston cores plotted in Figure 2c, 2d.

Thank you, we corrected this.

Line 370, Figure 4: Replace “e” with “f “in the figure caption

Thank you, we corrected this.

Methods: Line 552: How many samples in total were analyzed?

We measured 1593 samples for benthic oxygen isotope composition. We added this information to methods section.

Mean sortable silt and Ln(Zr/Rb) raw data from the three piston cores used for calculation of ACC strengths at these sites are missing. A figure similar to Extended Data Fig. 7 is needed in the supplementary material.

We agree and added such figures to Extended Data Fig. 8-10.

Similarly, you should add an additional figure in the supplementary material showing the XRF- Ca counts, discrete biogenic opal and calcium content measurements for the studied cores.

These data are now shown in Extended Data Fig. 8-10.

Line 676: The correlation between mean SS and Ln(Zr/Rb) shown in Extended Fig Data 9b is rather weak (0.42), compared to Wu et al., 2020 (0.83).

We agree to this point (also raised by Referee #2) that the correlation of ln(Zr/Rb) and sortable silt is relatively low, compared to previous studies. Most of the prior studies were located at or relatively proximal to continental margins with mainly siliciclastic sediment composition. This makes grain-size measurements less susceptible to incomplete removal of carbonates and biogenic opal (opal contents are generally low in most studies). This strongly differs from our setting with carbonate and opal sedimentation predominating. Therefore, we suspect that part of the scatter and the resulting relatively low correlation coefficient might be related to incomplete removal of biogenic components (though we controlled removal of carbonate and opal with the microscope). Further scatter might be introduced by the laser-particle sizes methods applied, because the remaining siliciclastic fraction was too small to use a Sedigraph which would be the preferred analytical instrument as it considers the dynamical behaviour of the sediment grains (McCave et al., 2006).

In line 619-621: You mention that this might be explained by a different composition of siliciclastic material in the SS fraction. Could you provide more details?

Yes, the Zr/Rb ratio in the source material may be different at continental margins compared to open ocean settings of the current study. This is based on the lower slope that we observe compared to other studies. We are not aware of Zr/Rb measurements in source regions which are difficult to define in the open ocean.

Extended Data Fig. 9a: Add units and values of ln(Zr/Rb) in the right side of the figure.

Thank you, we corrected this.

Reviewer Reports on the First Revision:

Referees' comments:

Referee #1 (Remarks to the Author):

Thank you to the authors for their detailed replies. Most of my comments have been addressed. There are a few points that remain.

1)Perhaps my first concern regarding how representative the records are of surface and column-integrated ACC strength did not come across clearly. These are bottom current records, so the authors should briefly acknowledge in the paper the potential caveat that changes in large-scale baroclinic density gradients across the Southern Ocean can lead to changes in the degree of vertical shear within the ACC. A subsequent question that comes up is if the sortable silt proxy is recording changes in both the mean and eddy components of the flow? If it is also recording changes in the eddy component then this could strengthen the connection with surface changes.

2)The framing “altered the sensitivity of the ACC from primarily atmospheric to increasingly oceanic forcings” (Ln 60-61) and “Taken at face value, this would imply a long-term strengthening of the ACC because increasing SST and atmospheric temperature gradients would strengthen the SWW driving the ACC.” (Ln 211-213) – not sure I agree with the "primarily atmospheric" framing, for ACC strengthening with Pliocene cooling both atmospheric and oceanic forcings were likely at play. The strengthening of SST gradients would also affect the density gradients across the Southern Ocean (oceanic forcing), as the authors state on Lns 234-236, this is an ocean forcing acting to strengthen the ACC across the Pliocene. It seems to me that a key argument the authors are trying to make is that there is a shift from global cooling = stronger Southern Ocean density gradients, SWW and ACC across the Pliocene, to global cooling = weaker Southern Ocean density gradients, equatorward shifted SWW and weaker ACC once Antarctic ice volume and sea-ice extent increases with the onset of iNHG. I am not sure the shift from primarily atmospheric to ocean framing helps get this point across.

3)On a related note, I found the “Long-term ACC development” section a bit hard to follow, and had to read it, and the review reply files, a few times to fully internalize the arguments being made. Some refinement, and perhaps a schematic illustrating the key conceptual mechanisms, is needed.

4)Ln 243-244 & Fig 4: Note that as with the Asian Monsoon response to modern El Nino events, the Pliocene East Asian Monsoon signal is heterogeneous across proxy records from different regions (see refs below). Which region is most important for the jet mechanism?

Lu, J., Yang, H., Griffiths, M. L., Burls, N. J., Xiao, G., Yang, J., ... & Xie, S. (2021). Asian monsoon evolution linked to Pacific temperature gradients since the Late Miocene. Earth and Planetary Science Letters, 563, 116882.

Gai, C., Liu, Q., Roberts, A.P., Chou, Y., Zhao, X., Jiang, Z., Liu, J., 2020. East Asian monsoon evolution since the Late Miocene from the South China Sea. Earth Planet. Sci. Lett.530, 115960.

Wang, H., Lu, H., Zhao, L., Zhang, H., Lei, F., Wang, Y., 2019. Asian monsoon rainfall variation during the Pliocene forced by global temperature change. Nat. Commun.10, 5272.

Zhang, Y.G., Ji, J., Balsam, W., Liu, L., Chen, J., 2009. Mid-Pliocene Asian monsoon intensification and the onset of Northern Hemisphere glaciation. Geology 37, 599–602.

An, Z., Kutzbach, John E., Prell, Warren L., Porter, Stephen C., 2001. Evolution of Asian monsoons and phased uplift of the Himalaya–Tibetan Plateau since Late Miocene times. Nature 411, 62–66.

5) Ln 256-260, I suggest removing this as this mechanism is discussed in the next section and just seems to muddy the waters when it comes to getting the key points of this section across.

Minor:

Ln 64-65: I suggest removing “On orbital timescales” as the previous sentence is already talking about orbital timescales.

Referee #2 (Remarks to the Author):

Review of resubmission of Lamy et al, ‘5 Ma of ACC strength’.
The data presented here are of good quality and provide a unique insight into ACC behaviour in a region where the ACC is unbounded topographically. Additionally the record is much longer than any others in the Southern Ocean.

The authors response to the potential difficulty with the relationship between Zr/Rb and silt size related to a possible weathering cause is satisfactory, calling on distance from a terrigenous source, but mention that this difficulty has been considered should be made in the Methods section.

Given a secure database the problem at hand is then attribution of the causes of variability in ACC strength over this period.
Pleistocene changes are clear and well set out with obvious maxima during warm periods.

However there are some problems in the authors’ attribution of causes of long-term ACC development. Figure 4c shows the SST difference between two sites about 2000 miles apart along the equatorial Pacific. (It is not clear what the relevance of a temperature difference along the equator is to the current problem concerning the ACC.) It is claimed on lines 213-4 that there is an ACC strength increase during Pliocene cooling where we are referred to figure 4. However over the period in question, 5-3.75 Ma, the

only temperature data in figure 4 is in 4c where it is constant, and the ice volume in 4b is also constant. On the basis of the contrast in supposed trends over this period and the ensuing period between 3250 and 1500 ka the authors deduce two fundamentally different modes of forcing. This is not strongly supported by the data at hand.

The opal data interpreted in lines 223 to 232 might corroborate the work by Lawrence, Hillenbrand, Burls and Haug (refs 32 to 35) but on their own are not a sufficient basis for deducing a strong meridional redistribution of Pacific nutrients. This section would be better indicated as a corroboration of the work by previous authors.

The argument that oceanic fronts were weakly developed during the Pliocene and that increasing wind stress was responsible for the general increase in flow strength between 5.25 and 3.25 Ma seems well founded.

In the first paragraph of the section on Orbital Forcing it would be helpful to refer to extended figure 11 where the 400 ka filter of ACC strength and other components are shown.

Is another interpretation of lines 287 to 290 that, perhaps, the connection between the ACC and the monsoon is really very weak?

The anti-phase relationship between eccentricity and carbon isotopes in the Pliocene and early Pleistocene is very clear, but can the authors explain the sequence of events – eccentricity forcing relationship to carbon isotopes - from 1500 ka to the present?

The section on relationship between ACC strength and the Antarctic ice sheet is entirely credible and important.

The evidence, from this segment of the ACC at least, supports the argument that warming climate should lead to a strengthened ACC.

References.
Rarely can such an error-strewn set of references have been submitted to a major journal. Specifically:
1. ARTN (or artn) followed by a number (maybe to someone's reference database) should not be in any DOI (refs 2, 6, 8, 10, 19, 22, 23, 34, 43, 44, 47).
2. Proper page numbers should be included not simply the first page and a '+'; (refs 9, 17, 18, 30, 31, 45).
3. DOI is doubled in several places, refs 20, 35, 39, 54.
4. Incorrect capitalisation occurs in refs 3, 8, 15,19, 29, 37, 40
5. references 56 and 50 should probably refer to Proceedings of the ODP, 383 (as in reference 24).
6. Meaningless strings of letters (?initials?)in 24, 50, 56.

Doubtless there are more errors, but these are the most obvious ones. You would have thought that with 38 authors at least one could have been deputed to check the references.

Referee #3 (Remarks to the Author):

Review of the manuscript " Five million years of Antarctic Circumpolar Current strength variability " by Lamy and co-authors

I appreciate the thought, time and effort of the authors have put into addressing the concerns raised during the first review process. As mentioned in my previous review, this manuscript presents a great set of new data, and will provide an important contribution to the field. However, I still have some concerns on some of the interpretations and mechanistic links proposed by the authors.

“Pleistocene ACC strength changes” section

Although the authors describe clearly their data in this section, I still find their interpretation not robust. I miss the main message of this section. The authors propose that the observed fluctuations of ACC strength and opal content might reflect strengthening of the opal belt - that do not necessarily require a substantial latitudinal shift of the opal belt, but may imply changes in upwelling and ocean stratification iron fertilization. I am not completely against this interpretation, but I need to see evidence for that. I am saying that because an alternative explanation of these fluctuations could be that, the observed glacial-interglacial patterns during Pleistocene may result of latitudinal migrations of the ACC frontal system. Thus, the ACC strength varies not because the ACC flow gets weaker or stronger as a whole, but because the SAF/PF moves away (north or south) of the core locations.

Looking at the opal and $CaCO_3$ % data in Extended Data Figs 8-10, and the strong anti-correlation between them, I am more convinced that the data actually reflect latitudinal changes of the frontal system, which ultimately influence your SS data.

Moreover, the authors propose a link between the high opal content and stronger upwelling, without substantially latitudinal shifts (lines 199-202). Wouldn’t they expect to record stronger ACC speeds then? The authors actually refer to this mechanism in lines 271-273, 295-298 and in the “ACC strength and Antarctic Ice sheets” section.

“Long-term ACC development” section

I find interesting the way that the authors argue for the contrasting trends on their data between the middle-late Pliocene and the early Pleistocene. However, I am not yet convinced on their compensating mechanism for the early Pleistocene, especially their arguments between lines 254-256 “After this time … during the Pliocene cooling.” The authors need to back up this with data and/or model studies from the literature, that show a degreasing meridional gradients in the Southern mid-latitudes. For example,

Hill et al. (2017 -Nature Communications) show an increase in winds and wind stress on the Southern Ocean resulting in a stronger ACC during the Plio-Pleistocene transition, driven by Antarctic ice advance. This model results contrast the proposed mechanism.

Minor comments:

Line 58-60: “Pliocene global cooling”, “early Pleistocene cooling”, “Late Pliocene Southern Ocean reconfiguration”. You may want to add some ages in a parenthesis. This will help the reader.

Line 65: “Equatorward extended opal deposition”, what do you mean by “extended” opal deposition?

Lines: 183- 186: What does this comparison actually show? I think you miss a final sentence there.

Line 313: “…sheet development during the Pliocene”. Why only Pliocene?

Figure 3d: “Early MPT ACC strengthening”
Fig. 4b: Nasih-> Naish.

Fig. 4b: Describe in the figure caption what is the blue, green and yellow intervals you show.

Methods: I cannot find the core names and mean SS values you used to calculate the Holocene mean. Please provide this information.

Author Rebuttals to First Revision:

Response to Referees

Response to Referee #1

(comments by the reviewer are printed in italics, our point-by-point response in blue)

Thank you to the authors for their detailed replies. Most of my comments have been addressed. There are a few points that remain.

1)Perhaps my first concern regarding how representative the records are of surface and column-integrated ACC strength did not come across clearly. These are bottom current records, so the authors should briefly acknowledge in the paper the potential caveat that changes in large-scale baroclinic density gradients across the Southern Ocean can lead to changes in the degree of vertical shear within the ACC. A subsequent question that comes up is if the sortable silt proxy is recording changes in both the mean and eddy components of the flow? If it is also recording changes in the eddy component then this could strengthen the connection with surface changes.

We thank Referee #1 for bringing up potential caveats in interpolating our bottom current proxy records and their significance for ACC strength considering the modern oceanography of the ACC. We have followed the reviewer's suggestion to expand on our discussion of the ACC dynamics and proxy interpretation.

In the revised manuscript, we extend our general introduction to the major controlling factors of the modern ACC at the beginning of the main text (lines 69 to 85), building on the fundamental work of the modern forcings of the ACC by Rintoul et al. (2018; Nature) and the significance of the sortable silt proxy for past ACC reconstructions given in the paper by McCave et al., (2014, Nat. Geo). Specifically, we include the significance of baroclinic density gradients, eddy field variations and "eddy saturation" respect to the wind forcing of the ACC. The adjustment time-scale for changing the density structure across the ACC lies in the range of decades or longer, limiting any variations in ACC transport on short time-scales. However, our sortable silt-based data (recording near bottom currents) document ACC variations on much longer time-scales. For example, even our high resolution core-scanning (Zr/Rb data) records still average across centuries. McCave et al. (2014) stress that the sediment-derived near bottom current records are scalar mean current speeds that include the eddy component.

Therefore, at our "paleocenaographic" time-scales, we conclude that sortable silt changes generally correspond to the total water transport including wind, baroclinic, and eddy-induced. Our sediment data therefore include both wind forcing and buoyancy changes within the ACC. We clarified this particular proxy implications for the ACC in the main text (lines 130 to 137).

2)The framing "altered the sensitivity of the ACC from primarily atmospheric to increasingly oceanic forcings" (Ln 60-61) and "Taken at face value, this would imply a long-term strengthening of the ACC because increasing SST and atmospheric temperature gradients would strengthen the SWW driving the ACC." (Ln 211-213) – not sure I agree with the "primarily atmospheric" framing, for ACC strengthening with Pliocene cooling both atmospheric and oceanic forcings were likely at play. The strengthening of SST gradients would also affect the density gradients across the Southern Ocean (oceanic forcing), as the authors state on Lns 234-236, this is an ocean forcing acting to strengthen the ACC across the Pliocene. It seems to me that a key argument the authors are trying to make is that there is a shift from global cooling = stronger Southern Ocean density gradients, SWW and ACC across the Pliocene, to global cooling = weaker Southern Ocean density gradients, equatorward shifted SWW and weaker ACC once Antarctic ice volume and sea-ice extent increases with the onset of iNHG. I am not sure the shift from primarily atmospheric to ocean framing helps get this point across.

We agree with the Reviewer that our framing of "predominantly" atmospheric versus oceanic forcing might be too simplified. Referee #1 is correct that both atmospheric and oceanic forcings operate and interact, including during the Pliocene. We now better outline the principal differences in atmospheric and oceanic forcings of the ACC for the Pliocene versus the Pleistocene long-term trends. We therefore rephrased the respective text in the abstract (lines 58 to 60) and the "Long-term ACC development" section and further stress the different impact of global cooling on Southern Ocean density gradients for both time intervals (lines 217-289).

3)On a related note, I found the "Long-term ACC development" section a bit hard to follow, and had to read it, and the review reply files, a few times to fully internalize the arguments being made. Some refinement, and perhaps a schematic illustrating the key conceptual mechanisms, is needed.

We agree with the reviewer's comment. We clarified our arguments at several locations in the text and largely rewrote the "Long-term ACC development" section (lines 217-289). We have followed the reviewer's suggestion and also now illustrate our concepts schematically (New Fig. 5)

4)Ln 243-244 & Fig 4: Note that as with the Asian Monsoon response to modern El Nino events, the Pliocene East Asian Monsoon signal is heterogeneous across proxy records from different regions (see refs below). Which region is most important for the jet mechanism?

Lu, J., Yang, H., Griffiths, M. L., Burls, N. J., Xiao, G., Yang, J., ... & Xie, S. (2021). Asian monsoon evolution linked to Pacific temperature gradients since the Late Miocene. Earth and Planetary Science Letters, 563, 116882.

Gai, C., Liu, Q., Roberts, A.P., Chou, Y., Zhao, X., Jiang, Z., Liu, J., 2020. East Asian monsoon evolution since the Late Miocene from the South China Sea. Earth Planet. Sci. Lett.530, 115960.

Wang, H., Lu, H., Zhao, L., Zhang, H., Lei, F., Wang, Y., 2019. Asian monsoon rainfall variation during the Pliocene forced by global temperature change. Nat. Commun.10, 5272.

Zhang, Y.G., Ji, J., Balsam, W., Liu, L., Chen, J., 2009. Mid-Pliocene Asian monsoon intensification and the onset of Northern Hemisphere glaciation. Geology 37, 599–602.

An, Z., Kutzbach, John E., Prell, Warren L., Porter, Stephen C., 2001. Evolution of Asian monsoons and phased uplift of the Himalaya–Tibetan Plateau since Late Miocene times. Nature 411, 62–66.

We thank Referee #1 for highlighting the regional differences of Pliocene paleo monsoon records as documented in the references mentioned. We agree that the modern East Asian Summer Monsoon (EASM) response to ENSO and the proxy evidence for Pliocene changes varies across the EASM region. In our response, we follow the most recently published paper by Lu et al., (2021 EPSL). Lu et al review the geographic pattern of EASM changes in terms of a meridional tripole pattern with overall increased rainfall in northern and southern China paralleling dryer conditions in central China.

In our manuscript, we decided to compare our data to the Chinese Loess Plateau record from the Lingtai Section (Sun et al., 2010 EPSL) located in the northern zone of the tripole. The Lingtai Section provides a well dated (based on orbital tuning and paleomagnetics) proxy record of summer monsoon strength using primarily magnetic susceptibility changes. Compared to other loess proxies (e.g. carbonate content), the magnetic susceptibility changes in the Mio-Pliocene Red Clay formation of the Lingtai Section document

the long-term EASM evolution during the Pliocene, in contrast to carbonate content fluctuations, reflecting more clearly orbital-scale variations (Sun et al., 2010 EPSL).

Lu et al. (2021 EPSL) provide also evidence that interannual (e.g. ENSO) pattern can be extrapolated to Pliocene monsoon changes, as provided by both proxy and modelling evidence (e.g. Burls et al., 2017). In this context, we discuss the long-term increase in the Lingtai magnetic susceptibility record, indicating increasing EASM from ~4.2 to 2.75 Ma connected to the initial development of zonal SST gradients across the tropical Pacific. This trend corresponds to an overall change from El Niño-like climate pattern in the early Pliocene towards increasingly La Nina-like conditions in the late Pliocene. Modelling studies by Lu et al. (2021 EPSL) and Burls et al. (2017; Sci. Adv.) strengthen the application of the EASM tripole to Pliocene paleoclimates. For example, the expanded Western Pacific Warm Pool and weaker zonal and meridional temperature gradients during the early Pliocene results in reduced northward transport of monsoonal moisture (e.g. to the Lingtai site) but increased convergence in the South China Sea leading to higher rainfall in central-eastern China as shown in other records (e.g., Gai et al., 2019; EPSL). We added more detail on the EASM in lines 243-253.

The large-scale changes in the “northern” EASM connected to zonal and meridional Pacific SST pattern has a strong influence on tropical and subtropical atmospheric circulation through affecting Hadley and Walker circulations from the early Pliocene towards the late Pliocene. The exact atmospheric teleconnections of these subtropical/tropical atmosphere-ocean changes to our reconstructed ACC fluctuations plausibly including changes in the southern westerly winds are difficult to constrain in more detail. As discussed in the manuscript, the Pleistocene South Pacific Split jet is linked to varying tropical Pacific zonal SST gradients and the EASM. During this time span, enhanced zonal SST gradients correspond to weakened EASM, a stronger split jet with reduced subantarctic westerly winds. This response is opposite to the proxy evidence for the Pliocene long-term trends and indicates that long-term Plio-Pleistocene ACC – tropical/subtropical linkages including the EASM operate differently. We updated this section in lines 311-325.

5*) Ln 256-260, I suggest removing this as this mechanism is discussed in the next section and just seems to muddy the waters when it comes to getting the key points of this section across.*

We agree and have integrated parts of this text to the next section.

Minor:

Ln 64-65: I suggest removing "On orbital timescales" as the previous sentence is already talking about orbital timescales.

We agree and have edited the text accordingly.

Referee #2 (Remarks to the Author):

Review of resubmission of Lamy et al, '5 Ma of ACC strength'.

The data presented here are of good quality and provide a unique insight into ACC behaviour in a region where the ACC is unbounded topographically. Additionally the record is much longer than any others in the Southern Ocean.

The authors response to the potential difficulty with the relationship between Zr/Rb and silt size related to a possible weathering cause is satisfactory, calling on distance from a terrigenous source, but mention that this difficulty has been considered should be made in the Methods section.

We added this information to the methods (lines 769-775).

Given a secure database the problem at hand is then attribution of the causes of variability in ACC strength over this period.

Pleistocene changes are clear and well set out with obvious maxima during warm periods.

We thank Referee #2 for this positive assessment.

However, there are some problems in the authors' attribution of causes of long-term ACC development. Figure 4c shows the SST difference between two sites about 2000 miles apart along the equatorial Pacific. (It is not clear what the relevance of a temperature difference along the equator is to the current problem concerning the ACC.) It is claimed on lines 213-4 that there is an ACC strength increase during Pliocene cooling where we are referred to figure 4. However over the period in question, 5-3.75 Ma, the only temperature data in figure 4 is in 4c where it is constant, and the ice volume in 4b is also constant. On the

basis of the contrast in supposed trends over this period and the ensuing period between 3250 and 1500 ka the authors deduce two fundamentally different modes of forcing. This is not strongly supported by the data at hand.

The Reviewer correctly noted that we do not refer to a more global temperature record for our Pliocene discussion. We mention the increasing zonal (Liu et al.) and meridional temperature gradients (Federov et al. 2015; Nat. Geo., Herbert et al., 2016; Nat. Geo.) in the text. In Figure 4, we have now replaced the cold-tongue zonal temperature gradient reconstruction by Liu et al. with the zonal and meridional temperature gradient reconstruction by Federov et al. (2015) (new Fig. 4c). These reference gradient reconstructions for the Pacific realm are well-suited to compare with our long-term ACC trends (lines 241-250) and support our mechanistic interpretation.

The opal data interpreted in lines 223 to 232 might corroborate the work by Lawrence, Hillenbrand, Burls and Haug (refs 32 to 35) but on their own are not a sufficient basis for deducing a strong meridional redistribution of Pacific nutrients. This section would be better indicated as a corroboration of the work by previous authors.

We thank Referee #2 for highlighting this section. We agree that the importance of the opal records might not have been put sufficiently in the context of the prior work on opal and nutrient distributions. We now restructured this paragraph in order to clarify the importance of the ACC for the meridional redistribution of Pacific nutrients from the North Pacific to the subantarctic Southern Ocean (lines 279-289).

The argument that oceanic fronts were weakly developed during the Pliocene and that increasing wind stress was responsible for the general increase in flow strength between 5.25 and 3.25 Ma seems well founded.

We thank the reviewer for this evaluation.

In the first paragraph of the section on Orbital Forcing it would be helpful to refer to extended figure 11 where the 400 ka filter of ACC strength and other components are shown.

We thank the reviewer for the comment and have added the reference to Extended Data Figure 9 (please note updated numbering).

Is another interpretation of lines 287 to 290 that, perhaps, the connection between the ACC and the monsoon is really very weak?

While it is difficult to evaluate the strength of teleconnections, we can use the amplitude, sign and direction of fluctuations of the ~400-kyr-filtered time series to deduce a relationship between the ACC and the EASM. We therefore hypothesize that the antiphasing between ACC strength and the monsoon variations is important and indicates long-term orbital changes that are not identical to the shorter orbital cycles, e.g. precessionial variations. To clarify this point, we have added an additional sentence to our discussion (lines 312-325), i.e. that long-term ACC-monsoon teleconnections operate differently and do not involve the South Pacific Split Jet. Please see also our response to Referee #1.

The anti-phase relationship between eccentricity and carbon isotopes in the Pliocene and early Pleistocene is very clear, but can the authors explain the sequence of events – eccentricity forcing relationship to carbon isotopes - from 1500 ka to the present?

We can only refer to the literature for explaining the middle and late Pleistocene changes in the carbon isotope phasing. The cited review paper by Wang et al., 2014 (National Science Review) notes that after ~1.6 Ma, the clear correlation Miocene to Pliocene correlation of oceanic $\delta^{13}C$ to eccentricity breaks down. The authors suggest, that ocean restructuring at ~1.6 Ma marked by the isolation of a sluggish abyss under the Southern Ocean that has obscured the long-eccentricity 400-kyr signal in oceanic $\delta^{13}C$. This reorganization of the oceanic carbon reservoir changes the response to the ~400-kyr-eccentricity forcing probably also connected to major changes related to the Mid-Pleistocene Transition and the mid-Brunhes event in the middle Pleistocene (Wang et al., 2014 and the original reference Wang et al., 2010 (EPSL).

The section on relationship between ACC strength and the Antarctic ice sheet is entirely credible and important.

We thank the reviewer for this evaluation.

The evidence, from this segment of the ACC at least, supports the argument that warming climate should lead to a strengthened ACC.

We again thank the reviewer for this evaluation.

References.

Rarely can such an error-strewn set of references have been submitted to a major journal. Specifically:

1. ARTN (or artn) followed by a number (maybe to someone's reference database) should not be in any DOI (refs 2, 6, 8, 10, 19, 22, 23, 34, 43, 44, 47).

2. Proper page numbers should be included not simply the first page and a '+'; (refs 9, 17, 18, 30, 31, 45).

3. DOI is doubled in several places, refs 20, 35, 39, 54.

4. Incorrect capitalisation occurs in refs 3, 8, 15,19, 29, 37, 40

5. references 56 and 50 should probably refer to Proceedings of the ODP, 383 (as in reference 24).

6. Meaningless strings of letters (?initials?)in 24, 50, 56.

Doubtless there are more errors, but these are the most obvious ones. You would have thought that with 38 authors at least one could have been deputed to check the references.

We apologize for the errors in the References. We have now made sure that the revised list is correct.

Referee #3 (Remarks to the Author):

Review of the manuscript " Five million years of Antarctic Circumpolar Current strength variability " by Lamy and co-authors

I appreciate the thought, time and effort of the authors have put into addressing the concerns raised during the first review process. As mentioned in my previous review, this manuscript presents a great set of new data, and will provide an important contribution to the field. However, I still have some concerns on some of the interpretations and mechanistic links proposed by the authors.

"Pleistocene ACC strength changes" section

Although the authors describe clearly their data in this section, I still find their interpretation not robust. I miss the main message of this section. The authors propose that the observed fluctuations of ACC strength and opal content might reflect strengthening of the opal belt - that do not necessarily require a substantial latitudinal shift of the opal belt, but may imply changes in upwelling and ocean stratification iron fertilization. I am not completely against this interpretation, but I need to see evidence for that. I am saying that because an alternative explanation of these fluctuations could be that, the observed glacial-interglacial patterns during Pleistocene may result of latitudinal migrations of the ACC frontal system. Thus, the ACC strength varies not because the ACC flow gets weaker or stronger as a whole, but because the SAF/PF moves away (north or south) of the core locations.

Looking at the opal and CaCO3 % data in Extended Data Figs 8-10, and the strong anti-correlation between them, I am more convinced that the data actually reflect latitudinal changes of the frontal system, which ultimately influence your SS data.

Moreover, the authors propose a link between the high opal content and stronger upwelling, without substantially latitudinal shifts (lines 199-202). Wouldn't they expect to record stronger ACC speeds then? The authors actually refer to this mechanism in lines 271-273, 295-298 and in the "ACC strength and Antarctic Ice sheets" section.

We thank the reviewer for pointing us to the interpretation of our opal and ACC strength data in terms of latitudinal frontal shifts versus ACC strength changes. The ACC strength records shown in Fig. 2 across the Pleistocene core transect from the SAZ to the AZ (including the shorter piston cores not reaching back to the earlier Pleistocene and Pliocene) show consistent glacial reductions of ACC strength across the SAF and PF with the largest decrease in the SAZ. In the original manuscript, we interpret these glacial-interglacial ACC changes as not necessarily requiring a large latitudinal shift of the ACC fronts. The reviewer is correct pointing out that the substantial glacial opal increases do indicate latitudinal shifts of the fronts, as we discuss later in relation to longer-term changes including Pliocene.

In the revised manuscript, we clarify our interpretation integrating both the ACC strength and the opal content data indicating frontal movements in combination with overall ACC strength changes at the end of the section discussing the Pleistocene transect (lines 187-201). Our data indicate that both mechanisms are important but it is not possible to separate the relative contributions.

Accordingly, we clarified the discussion regarding the significance of frontal movements for opal fluctuations on longer time-scales (lines 187-201).

"Long-term ACC development" section

I find interesting the way that the authors argue for the contrasting trends on their data between the middle-late Pliocene and the early Pleistocene. However, I am not yet convinced on their compensating mechanism for the early Pleistocene, especially their arguments between lines 254-256 "After this time … during the Pliocene cooling." The authors need to back up this with data and/or model studies from the literature, that show a degreasing meridional gradients in the Southern mid-latitudes. For example, Hill et al. (2017 -Nature Communications) show an increase in winds and wind stress on the Southern Ocean resulting in a stronger ACC during the Plio-Pleistocene transition, driven by Antarctic ice advance. This model results contrast the proposed mechanism.

Regarding the decrease of meridional temperature gradients, we referred to the proxy data-based long-term meridional and zonal temperature gradient reconstructions by Federov et al. (2015; Nat. Geo). In the revised manuscript, we now plot these gradients in Fig. 4c (replacing the previously shown cold tongue data by Liu et al.). These records show an overall long-term decrease of both gradients in the Pacific realm over the Plio-Pleistocene.

As pointed out by the reviewer, the modelling study by Hill et al., (2017 Nat. Comm.) mentions an increasing ACC strength when comparing Pliocene Model Intercomparison Project simulations (PlioMIP, focussing on the mid-Piacenzian Warm Period between 3.264–3.025 Ma)), to simulations imposing modern Antarctic Ice Sheet configurations (WAIS and EAIS) on the Pliocene model set-up. Hill et al. show a large increase of the southern part of the modern ACC and a substantial decrease of the near-shore Antarctic counter when imposing the Antarctic ice-sheets. They assume that these changes are primarily wind-driven.

These modelling results contrast our decreasing long-term trend in ACC strength associated with the iNHG as discussed in the paper (lines 263-268). Reasons for these proxy-records versus model simulations discrepancies are potentially manifold. One aspect, might be that Hill et al., primarily invoke atmospheric forcing and do not discuss frontal systems, density stratification, and others. Moreover, they base the simulation on the Pliocene warm setting imposing the Antarctic ice-sheet, whereas as our proxy data record the transient changes across the Plio-Pleistocene including high- and low latitude forcing.

We now mention the Hill et al. study in the manuscript indicating that the Late Pliocene/early Pleistocene WAIS advance has a strong impact on various aspects of Pacific circulation including the ACC. However, the sign of the ACC impact appears opposite of our decreasing ACC changes.

Minor comments:

Line 58-60: "Pliocene global cooling", "early Pleistocene cooling", "Late Pliocene Southern Ocean reconfiguration". You may want to add some ages in a parenthesis. This will help the reader.

Given the emphasis of the masnuscript on long-term trends, we have limited the references to specific aged to increase readability of the paper. At the same time, we have been careful to mark all the geological time periods extensively in the figures to provide context.

Line 65: "Equatorward extended opal deposition", what do you mean by "extended" opal deposition?

We originally used "extended opal deposition" as it is difficult to distinguish glacial/interglacial latitudinal shift of the fronts and thus opal belt, or a real extension, i.e. geographically larger glacial opal belt. The latter could be explained e.g. by higher iron fertilisation in the SAZ, combined with the frontal movements northward.

Lines: 183- 186: What does this comparison actually show? I think you miss a final sentence there.

We are sorry, this is not correctly formulated. We reworded this part.

Line 313: "...sheet development during the Pliocene". Why only Pliocene?

We confined our comparison to ANDRILL to the Pliocene as the Pleistocene section of the ANDRILL (AND-1B) ice-extent reconstructions is not continuous and cannot be used for detailed comparisons. We therefore added also the modelled sediment facies extending to the present but as these are model results,

we opted to not compare these in detail to our proxy record but rather limit our interpretation to the robust sections of the ANDRILL reconstruction from the Pliocene..

Figure 3d: "Early MPT ACC strengthening"

Thank you, has been reworded.

Fig. 4b: Nasih-> Naish.

Thank you, has been corrected.

Fig. 4b: Describe in the figure caption what is the blue, green and yellow intervals you show.

Thank you, we added this information.

Methods: I cannot find the core names and mean SS values you used to calculate the Holocene mean. Please provide this information.

We added this information to the Methods.

Reviewer Reports on the Second Revision:

Referees' comments:

Referee #1 (Remarks to the Author):

I have read through the latest round of review replies and the updated manuscript. The authors have address all of my previous concerns. Specifically, the "Long-term ACC development" section is much clearer with the edits to the text and addition of the Fig. 5 schematic. I recommend acceptance.

Referee #2 (Remarks to the Author):

Review of Lamy et al. #3
Commentary
On the matter of spatial aliassing by frontal shifts, it appears from the 0-350 ka data that the whole current increases and decreases across most of the fronts in concert as they shift between glacial and interglacial positions. The glacial to Holocene 5° latitudinal shifts are sufficient to move the glacial polar front into a similar position to the modern subantarctic front and there is no reason to suppose that similar magnitudes of frontal shifts did not occur in the past. I therefore think that the aliassing problem is less severe than one might suppose. However I think the opal changes are correctly attributed to shifts in frontal locations (Lines 213-4). One should also always bear in mind that we are dealing with order of hundred-year averages.

Lines
121 and 154-5. The abbreviations SAZ, PFZ and AZ are not defined at first use. Southern Ocean specialists will know what these mean but many paleoceanographers may well not. (SAZ - north of the SubAntarctic front, PFZ -between the SubAntarctic Front and the Polar Front, AZ - south of the Polar Front and north of the Southern ACC Front.)
265-6. When a model conflicts with the data we should go with the data and re-evaluate the model.
287-8. The 'stronger North Pacific carbonate deposition' is possibly due to lower rates of carbonate dissolution caused by more CO3-saturated bottom water rather than changes in nutrients and productivity.
561. iNHG (insert i)
567. I find this statement ('negative values indicate gradient reduction towards the Pliocene') rather confusing and think that a phrase 'negative values indicate gradient increase from Pliocene to Holocene' would be clearer, although the authors' wording is exactly that used by Federov et al. Time flows from the Pliocene to the Holocene, not backwards.

The references have been well corrected and now have only minor blemishes of capitalisation on lines 428, 436-7, 508 and 517 (all from AGU journals).

Figures
It is not clear why ODP 1096 is shown on Fig. 1 because it is not mentioned at all in the text. It should be removed.

Referee #3 (Remarks to the Author):

I have reviewed Lamy et al ‘s revised manuscript “Five million years of Antarctic Circumpolar Current strength variability” several times. I find that the text now reads logical and clearly represents the main message of the authors. The figures represent well their data and interpretations. I also find the new schematic figure 5 very helpful. Thus, I have only a few minor comments:

Please be careful when submitting your revised documents. I received two different merged files, where color coding is different in Fig. 2c and Fig. 2d.
1. File “456723_2_merged_1701178906” (wrong)
2. File: “456723_2_merged_1701362611” (correct).

I recommend to define the analytical error for the grain size analyses and velocity records in the methods and/or figure captions. This information is missing.

Line 244: “(Fig 4c and 4d)”. You refer to the record of Chinese Loess Plateau as Fig. 4d in the text. But, it is shown in Fig. 4e.

Line 553: Figure 3c caption: The core name (PS75/93) is different than in the figure and text (PS97/93).

Line 580: “Shown are…” There is something missing in this sentence.

Extended Data Fig 1 b: I cannot find what the yellow crosses represent. I suggest to remove them.

Author Rebuttals to Second Revision:
Response to Referees

Response to Referee #1

(comments by the reviewer are printed in italics, our point-by-point response in blue)

I have read through the latest round of review replies and the updated manuscript. The authors have address all of my previous concerns. Specifically, the "Long-term ACC development" section is much clearer with the edits to the text and addition of the Fig. 5 schematic. I recommend acceptance.

We thank Referee #1 for their positive evaluation.

Response to Referee #2

(comments by the reviewer are printed in italics, our point-by-point response in blue)

Review of Lamy et al. #3
Commentary

On the matter of spatial aliassing by frontal shifts, it appears from the 0-350 ka data that the whole current increases and decreases across most of the fronts in concert as they shift between glacial and interglacial positions. The glacial to Holocene 5° latitudinal shifts are sufficient to move the glacial polar front into a similar position to the modern subantarctic front and there is no reason to suppose that similar magnitudes of frontal shifts did not occur in the past. I therefore think that the aliassing problem is less severe than one might suppose. However I think the opal changes are correctly attributed to shifts in frontal locations (Lines 213-4). One should also always bear in mind that we are dealing with order of hundred-year averages.

We appreciate and share the reviewer´s assessment.

Lines 121 and 154-5. The abbreviations SAZ, PFZ and AZ are not defined at first use. Southern Ocean specialists will know what these mean but many paleoceanographers may well not. (SAZ - north of the SubAntarctic front, PFZ -between the SubAntarctic Front and the Polar Front, AZ - south of the Polar Front and north of the Southern ACC Front.)

We thank Referee #2 for the suggested improvements and now consistently introduce the abbreviations at their first occurrence.

265-6. When a model conflicts with the data we should go with the data and re-evaluate the model.

In principal, we agree with Referee #2 that the proxy records are the primary source of paleoclimate information, while models are likely to not capture the complete climate mechanisms. We have adjusted the wording of the sentence in question accordingly.

287-8. The ‘stronger North Pacific carbonate deposition’ is possibly due to lower rates of carbonate dissolution caused by more CO3-saturated bottom water rather than changes in nutrients and productivity.

We agree with the reviewer. We largely follow the arguments presented in Burls et al. 2007 (Sci. Adv.). Carbonate preservation is an important process for explaining the CaCO3 maxima and reflect maxima in deep North Pacific ventilation and Pacific Meridional Overturning Circulation. We reworded accordingly to include preservation.

561. iNHG (insert i)

Thank you, corrected.

567. I find this statement (‘negative values indicate gradient reduction towards the Pliocene’) rather confusing and think that a phrase 'negative values indicate gradient increase from Pliocene to Holocene’ would be clearer, although the authors’ wording is exactly that used by Federov et al. Time flows from the Pliocene to the Holocene, not backwards.

We appreciate the suggested rewording and caption to read ‘Increasing values indicate the gradient increase since the Pliocene relative to the Holocene gradient'.

The references have been well corrected and now have only minor blemishes of capitalisation on lines 428, 436-7, 508 and 517 (all from AGU journals).

We agree with the reviewer that the capitalisation in mentioned AGU publications are a bit irritating and changed them accordingly.

Figures
It is not clear why ODP 1096 is shown on Fig. 1 because it is not mentioned at all in the text. It should be removed.

The opal record from ODP Site 1096 is shown in Fig. 4h. But we agree to remove the site location on Fig. 1 and show only the new sites from this study and the “ANDRILL” site.

Response to Referee #3

(comments by the reviewer are printed in italics, our point-by-point response in blue)

Referee #3 (Remarks to the Author):

I have reviewed Lamy et al 's revised manuscript "Five million years of Antarctic Circumpolar Current strength variability" several times. I find that the text now reads logical and clearly represents the main message of the authors. The figures represent well their data and interpretations. I also find the new schematic figure 5 very helpful. Thus, I have only a few minor comments:

Please be careful when submitting your revised documents. I received two different merged files, where color coding is different in Fig. 2c and Fig. 2d.
1. File "456723_2_merged_1701178906" (wrong)

2. File: "456723_2_merged_1701362611" (correct).

We apologize, we only noted these errors shortly after resubmission and updated the files with the help of the editor.

I recommend to define the analytical error for the grain size analyses and velocity records in the methods and/or figure captions. This information is missing.

We agree and expanded the methods section accordingly. We added: "While the standard analytical error of the grain-size analyses to obtain sortable silt values are in the range of ±0.6µm (at 20 µm, see below), the exact error of the current speed calculations from current meter data is more difficult to assess as only few current meter and grain-size data are available. McCave et al.[27] estimated the standard error to be in the range of ±12.5%".

Line 244: "(Fig 4c and 4d)". You refer to the record of Chinese Loess Plateau as Fig. 4d in the text. But, it is shown in Fig. 4e.

We appreciate the reviewer's point about the mix-up of the panels and corrected the reference to Fig. 4 accordingly.

Line 553: Figure 3c caption: The core name (PS75/93) is different than in the figure and text (PS97/93).

Sorry for this typo, we corrected accordingly.

Line 580: "Shown are…" There is something missing in this sentence.

We reworded this sentence.

Extended Data Fig 1 b: I cannot find what the yellow crosses represent. I suggest to remove them.

We followed the reviewer suggestion and removed the yellow crosses.
